# FgSfl1 and Its Conserved PKA Phosphorylation Sites Are Important for Conidiation, Sexual Reproduction, and Pathogenesis in *Fusarium graminearum*

**DOI:** 10.3390/jof7090755

**Published:** 2021-09-14

**Authors:** Chen Gong, Junqi Huang, Daiyuan Sun, Daiying Xu, Yuqian Guo, Jiangang Kang, Gang Niu, Chenfang Wang

**Affiliations:** College of Plant Protection, Northwest A&F University, Xianyang 712100, China; gongchen@nwafu.edu.cn (C.G.); hjqninet@163.com (J.H.); Sdybhmx1117@163.com (D.S.); DaiyingXu@hotmail.com (D.X.); A18730281665@163.com (Y.G.); jiangangkang123@163.com (J.K.); ng18829349464@163.com (G.N.)

**Keywords:** protein kinase A, spontaneous suppressors, plant infection, sexual reproduction, phosphorylation

## Abstract

The fungal plant pathogen, *Fusarium graminearum*, contains two genes, *FgCPK1* and *FgCPK2*, encoding the catalytic subunits of cAMP-dependent protein kinase A. *FgCPK1* and *FgCPK2* are responsible for most of the PKA activities and have overlapping functions in various cellular processes in *F. graminearum*. The *cpk1 cpk2* double mutant was significantly reduced in growth, rarely produced conidia, and was non-pathogenic. In this study, we found that the *cpk1 cpk2* double mutant was unstable and produced fast-growing spontaneous sectors that were defective in plant infection. All spontaneous suppressor strains had mutations in *FgSFL1*, a transcription factor gene orthologous to *SFL1* in yeast. Thirteen suppressor strains had non-sense mutations at Q501, three suppressor strains had frameshift mutations at W198, and five suppressor strains had mutations in the HSF binding domain of FgSfl1. Only one suppressor strain had both a non-synonymous mutation at H225 and a non-sense mutation at R490. We generated the *SFL1* deletion mutant and found that it produced less than 2% of conidia than that of the wild-type strain PH-1. The *sfl1* mutant was significantly reduced in the number of perithecia on carrot agar plates at 7 days post-fertilization (dpf). When incubated for more than 12 days, ascospore cirrhi were observed on the *sfl1* mutant perithecia. The infection ability of the *sfl1* deletion mutant was also obviously defective. Furthermore, we found that in addition to the S223 and S559 phosphorylation sites, *FgSFL1* had another predicted phosphorylation site: T452. Interestingly, the S223 phosphorylation site was responsible for sexual reproduction, and the T452 phosphorylation site was responsible for growth and sexual reproduction. Only the S559 phosphorylation site was found to play an important role in conidiation, sexual reproduction, and infection. Overall, our results indicate that *FgSFL1* and its conserved PKA phosphorylation sites are important for vegetative growth, conidiation, sexual reproduction, and pathogenesis in *F. graminearum*.

## 1. Introduction

Fusarium head blight (FHB), caused by *Fusarium graminearum*, is one of the most important diseases of various cereal crops [1]. The pathogen overwinters on plant debris, and ascospores released from perithecia in the spring are the initial origins of infection [2]. The ascospores are discharged on flowering wheat which become the center of the infection. After the initial colonization, the pathogen spreads from the infection site to other florets and reduces grain quality [2,3,4]. In addition, *F. graminearum* produces harmful mycotoxins, such as deoxynivalenol (DON) and zearalenone. DON is also phytotoxic and an important virulence factor in the wheat head blight fungus [5,6,7].

As a key secondary messenger, cyclic adenosine monophosphate (cAMP) synthesized by adenylate cyclase plays a central role in the transduction of environmental stimuli to its downstream target cAMP protein kinase A (PKA) [8]. The PKA holoenzyme consists of two regulatory subunits and two catalytic subunits. The binding of cAMP with the regulatory subunits results in the detachment and activation of the catalytic subunits [9]. In *Magnaporthe* *oryzae*, the *CPKA* and *CPK2* genes encode catalytic subunits of PKA. Although *CPKA* and *CPK2* are dispensable for hyphal growth, the *cpkA* mutant is delayed in appressorium formation and defective in appressorium turgor generation and, therefore, plant penetration. However, the *cpkA* mutant still forms appressoria on hydrophilic surfaces in response to exogenous cAMP [10]. In the budding yeast, three genes encode PKA catalytic subunits (*TPK1*, *TPK2*, and *TPK3*) and the triple mutant is non-viable [11]. The fission yeast has only one PKA catalytic subunit gene (*PKA1*), which is important but not essential for normal growth [12]. In the human pathogen *Aspergillus fumigatus*, the *pkaC1 pkaC2* double mutant is delayed in conidium germination in response to environmental nutrients and is significantly reduced in virulence [13]. In *F. graminearum*, the deletion of both *CPKA* and *CPK2* results in severe defects in growth and conidiation, and the double mutant is sterile in sexual reproduction and is nonpathogenic [14]. In *Ustilago maydis*, the *adr1 uka1* double mutant is defective in yeast growth, mating, and plant infection [15].

In *Saccharomyces cerevisiae*, *Sfl1* is one of the downstream transcription factors of the cAMP-PKA pathway, and its repressor function is negatively regulated by the major PKA catalytic subunit *Tpk2* [16]. In *M. oryzae*, the *cpkA cpk2* mutant is unstable, and spontaneous suppressor mutations often become visible in cultures more than 10 days old. *MoSFL1* can rescue growth defects in the *cpkA* *cpk2* double mutant. Interestingly, despite the slow growth rate, the *cpkA cpk2* double mutant can be recovered but is non-pathogenic. The deletion of *MoSFL1* has no effect on vegetative growth but results in reduced virulence and heat tolerance. Although MoSfl1 has unknown protein motifs in its C-terminal, the C-terminal region is essential for its negative regulatory function, because the C-terminal region of MoSfl1 is important for its interaction with MoCyc8. The deletion in the C-terminal of *MoSFL1* in the *cpkA cpk2* double mutant has the same phenotype as strains deleted in the *MoSFL1* in the *cpkA cpk2* double mutant. *MoSFL1* has three PKA phosphorylation sites in *M. oryzae* (S211, T441, and S554). Importantly, only S211 suppressed the defects in growth but not the appressorium formation of the *cpkA cpk2* double mutant [17].

In this study we collected 25 suppressor strains from the *cpk1 cpk2* double mutant of *F. graminearum*. We selected three suppressors for whole-genome sequencing and all three suppressors were mutated in *FgSFL1*. The deletion of *FgSFL1* affected conidiation and delayed sexual development. In wheat head infection assays, the disease index was reduced almost 50% compared to the wild type. Based on the features of the PKA phosphorylation site sequences [S/R] [S/R]-X-[S/T], we discovered another putative PKA phosphorylation site, T452, in addition to the two consensus PKA phosphorylation sites, S223 and S559. Three phosphorylation sites were responsible for the growth, sexual reproduction, and infection progress though precise regulation of phosphorylation in *F. graminearum*. These data indicate that *FgSFL1* and its phosphorylation sites play important roles in conidiation, sexual reproduction, and infection.

## 2. Experimental Procedures

### 2.1. Spontaneous Suppressors of the cpk1 cpk2 Mutant

Fast-growing sectors of the *cpk1 cpk2* mutant were transferred with sterile toothpicks to fresh oatmeal agar plates. After single-spore isolation, each subculture of spontaneous suppressors was assayed for defects in growth, conidiation, and plant infection [17].

### 2.2. Strains and Culture Conditions

The wild-type strain PH-1 [18] and mutants of *F. graminearum* generated in this study are listed in Table 1. Growth rate on potato dextrose agar (PDA) plates and race tubes with the PDA medium (data not shown), conidiation in a liquid CMC medium, and sexual reproduction on carrot agar plates were assayed as described previously [19,20]. Protoplast preparation and transformation of *F. graminearum* were performed as described [21]. Transformants were selected with 250 µg/mL hygromycin B (CalBiochem, Merck, KGaA, Darmstadt, Germany), 250 µg/mL geneticin G418 (Sigma, Burlington, MA, USA), or 200 µg/mL zeocin (Invitrogen, Burlington, MA, USA) in the top agar. For DNA isolation, vegetative hyphae were harvested by filtration from liquid YEPD (1% yeast extract, 2% peptone, 2% glucose) after incubation at 25 °C for 12 h.

### 2.3. Generation of Fgsfl1 Deletion Mutant of the cpk1 cpk2 Double Mutant

To generate the *cpk1 cpk2 Fgsfl1* mutant, the upstream and downstream flanking sequences of *FgSFL1* were amplified with primer pairs FgSFL1/1F-FgSFL1/2R (ble) and FgSFL1/3F (ble)-FgSFL1/4R (Table S1), respectively, and fused with the *ble* cassette amplified from pFL6 by overlapping PCR. Putative *cpkA cpk2 sfl1* mutants were screened by PCR with primers FgSFL1-7F and FgSFL1-8R. For DNA isolation, vegetative hyphae were harvested by filtration from liquid YEPD (1% yeast extract, 2% peptone, 2% glucose) after incubation at 25 °C for 12 h.

### 2.4. Generation of the sfl1 Mutant

For generating the gene replacement construct by the split-marker approach, the 562-bp upstream and 666-bp downstream fragments of *SFL1* were amplified with primer pairs FgSFL1/1F-FgSFL1/2R and FgSFL1/3F-FgSFL1/4R (Table S1), respectively. The resulting PCR products were connected to the *hph* hygromycin phosphotransferase cassette by overlapping PCR and then transformed into protoplasts of PH-1 as described [20]. Hygromycin-resistant transformants were screened by PCR with primers FgSFL1/5F and FgSFL1/6R (Table S1). They were further confirmed by a Southern blot analysis with its downstream flanking sequence as the probe.

For complementation assays, the entire *SFL1* gene and its promoter and terminator sequences were amplified with primers FgSFL1/CF and FgSFL1/CR2 (Table S1), digested with *Sma*I and *Sac*II, and cloned into the *NEO*^R^ vector pHZ100 [22]. The resulting construct pSFL1 was transformed into protoplasts of the SF-1. The *sfl1/SFL1* transformants were verified by PCR.

### 2.5. Plant Infection and DON Production Assays

For infection of wheat cultivar Xiaoyan 22, conidia of PH-1 and mutant strains were harvested from CMC cultures by filtration and re-suspended at 10^5^ spores/mL as described [7]. For each flowering wheat head, the fifth spikelet from the base was inoculated with 10 μL of conidial suspensions as described [19,23]. Spikelets with typical symptoms were examined 14 days post-inoculation (dpi) to estimate the disease index [24] and diseased wheat kernels were assayed for DON [22]. For each strain, plant infection and DON production assays were repeated at least three times.

### 2.6. Sexual Reproduction Assays

For self-fertilization, cultures were grown on carrot agar plates [25] for 7 days before pressing down aerial hyphae with a sterile 0.1% Tween-20 solution as described [26]. Perithecium formation, ascus development, and cirrhus production were assayed after incubation at 25 °C under black light for 1 to 2 weeks after fertilization [14,27].

### 2.7. Generation of FgSFL1^S223D^, FgSFL1^T452D^, and FgSFL1^S559D^ Transformants of SF-1

To generate the *FgSFL1*^S223D^, *FgSFL1*^T452D^, and *FgSFL1*^S559D^ constructs, we used a designable primer to amplify fragments (Table S1) and clone into pFL2 by the yeast gap repair approach [20,28]. The resulting *FgSFL1*^S223D^, *FgSFL1*^T452D^, and *FgSFL1*^S559D^ constructs were confirmed by a sequencing analysis and transformed into protoplasts of SF-1. Transformants expressing the *FgSFL1*^S223D^, *FgSFL1*^T452D^, and *FgSFL1*^S559D^ constructs were analyzed by PCR.

### 2.8. Generation of FgSFL1^S223A^, FgSFL1^T452A^, and FgSFL1^S559A^ Transformants of SF-1

Refer to the above method.

### 2.9. RNA-Seq Analysis

A total of 12 h germlings of the wild-type strain PH-1 and *Fgsfl1* mutants were collected to isolate RNA (two biological replicates each). The RNA sample was sequenced with Illumina HiSeq 2500 with the paired-end 2 × 150 bp model at the Novogene Bioinformatics Institute (Beijing, China). All obtained RNA-seq reads (at least 24 Mb each sample) were mapped onto the reference genome of *F.* *graminearum* wild-type strain PH-1 [18,29] by HISAT2 [29]. In addition, RNA-seq data were deposited in the NCBI SRA database under the accession number SRR15569465 and SRR15569464. All reads mapped to each gene were calculated by featureCounts [30]. Differentially expressed genes with the false discovery rate (FDR) < 0.05 and log2 fold change (Log2FC > 1) were identified with the edgeRun package as described [31].

### 2.10. qRT-PCR Analysis

A total of 12 h germlings of the wild-type strain PH-1 and *Fgsfl1* mutants were collected to isolate RNA (two biological replicates each). The RNA sample was isolated by Eastep Super Total RNA Extraction Kit (Promega, Madison, WI, USA). Reverse transcription RNA used the HiScript II One Step qRT-PCR Probe Kit (Vazyme, Nanjing, China). The qRT-PCR test used the ChamQ SYBR qPCR Master Mix (Vazyme, China). All qRT-PCR data are in the Supplementary File Figure S8.

## 3. Results

### 3.1. The CPK1 CPK2 Mutant Is Unstable and Produces Spontaneous Suppressor Strains with Faster Growth Rate

**Figure 1:**
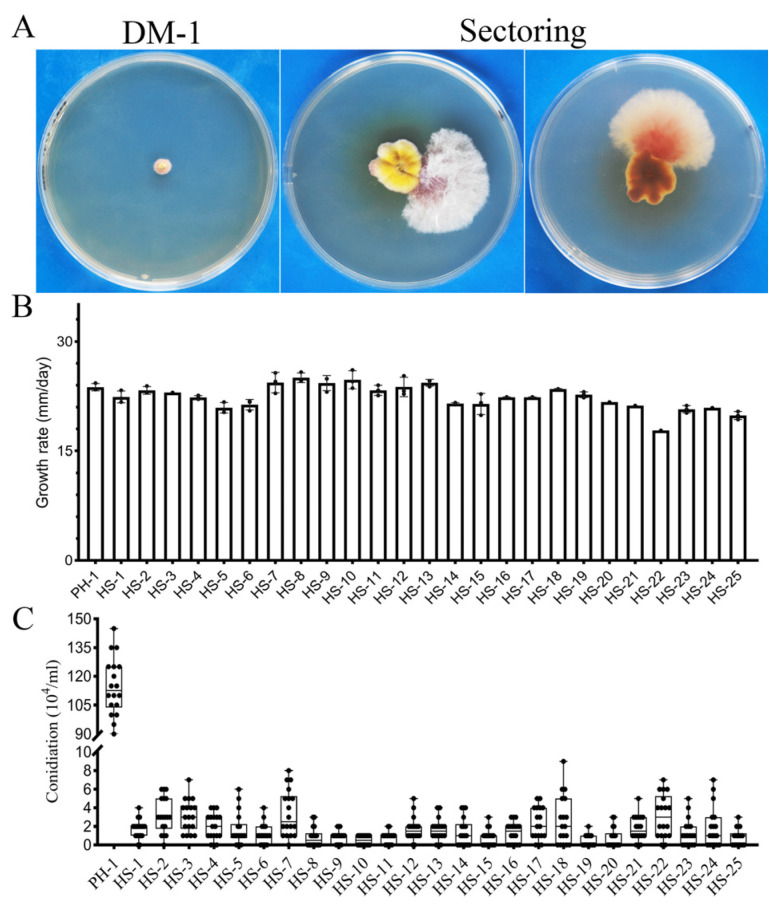
Spontaneous suppressors of the cpk1 cpk2 double mutant. (**A**) Three-day-old PDA cultures of the *cpk1 cpk2* double mutant and *cpk1 cpk2* double mutant with fast-growing sectors. (**B**) Assay of growth rate of the spontaneous suppressor strains. Mean and standard errors were estimated with data from three independent measurements. (**C**) Assay of conidiation of the spontaneous suppressor strains. Mean and standard errors were estimated with data from three independent measurements.

The *cpk1 cpk2* double mutant strain DM-1 was unstable when cultured on PDA at 25 °C. Fast-growing sectors derived from spontaneous mutations often became visible after incubation for 12 days or longer (Figure 1A). We randomly collected 25 subcultures of spontaneous sectors. Whereas five of them (20%) had a similar growth rate to the wild-type strain PH-1, five (20%) sectors grew faster than the wild-type strain PH-1. All the other suppressor strains grew faster than the original mutant but slightly slower than the wild type (Figure 1B and Figure S1). Nevertheless, like the *cpk1 cpk2* mutant, all the suppressor strains were still defective in conidiation (Figure 1C). These results indicate that spontaneous mutations in these suppressor strains rescued vegetative growth but did not positively impact conidiation.

### 3.2. Suppressors HS-20 and HS-25 Restored Some Asexual and Sexual Reproduction Defects of the cpk1 cpk2 Double Mutant

**Figure 2:**
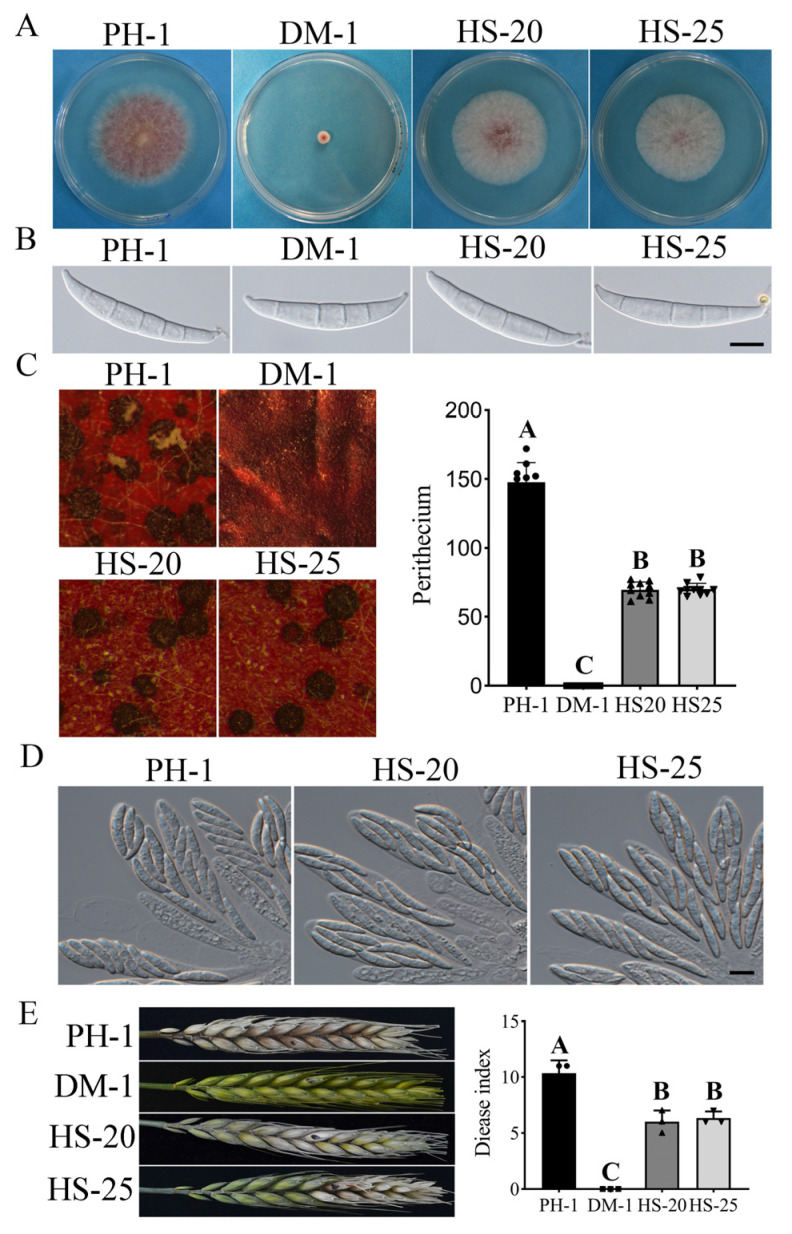
Spontaneous suppressor strains recovered in growth rate, sexual development, and virulence. (**A**) Three-day-old PDA cultures of the wild-type strain PH-1, DM-1, HS-20, and HS-25. (**B**) Conidiation of the wild-type strain PH-1, DM-1, HS-20, and HS-25 after cultivation in CMC for five days. Bar = 20 µm. (**C**) Carrot agar cultures of PH-1, DM-1, HS-20, and HS-25 were examined 7 days post-fertilization (dpf) and assay of perithecia of the wild-type strain PH-1, DM-1, HS-20, and HS-25. (**D**) Ascospores of the wild-type strain PH-1, HS-20, and HS-25 were examined by differential interference contrast (DIC). Bar= 10 µm. (**E**) Flowering wheat heads were drop-inoculated with conidia of the same set of strains and photographed 14 days post-inoculation (dpi). Black dots mark the inoculated spikeletes. Different letters indicate significant differences based on ANOVA analysis followed by Duncan’s multiple range test (*p* = 0.01) in A, B, C.

We selected suppressor strains HS-20 and HS-25 (Table 1) for further characterization because their growth rate was 98% and 96% of the wild-type strain PH-1, respectively (Table 2; Figure 2A). Although conidium morphology was not affected (Figure 2B), strains HS-20 and HS-25 were significantly reduced in conidiation (Figure 1C; Table 2). On self-mating carrot agar plates, the wild-type strain PH-1 produced abundant perithecia and ascospore cirrhi. The *cpk1 cpk2* double mutant was sterile and failed to develop perithecia at 7 days post-fertilization (dpf) or longer. Under the same conditions, suppressor strains HS-20 and HS-25 produced fewer perithecia than the wild type (Figure 2C) and no cirrhi were observed, but they produced normal ascospores (Figure 2D). Upon continued incubation of HS-20 and HS-25, abnormal white cirrhi were observed from 12-dpf to 14-dpf (Figure S2A). These results show that the suppressor strains partly restore the sexual reproduction but not the ascospore discharge of the *cpk1 cpk2* double mutant.

In infection assays with flowering wheat heads, the wild-type strain PH-1 caused typical head blight symptoms at 14 days post-inoculation (dpi). The *cpk1 cpk2* double mutant caused typical symptoms only on the inoculated kernels and failed to spread to neighboring spikelets (Figure 2E). The suppressor strains HS-20 and HS-25 caused typical symptoms from the vaccination point to neighboring spikelets with a disease index that was 59% and 60.7% that of the wild type (Table 2). These results suggest that mutations in the suppressor strains HS-20 and HS-25 not only suppressed the defects of the *cpk1 cpk2* double mutant in hyphal growth but also in sexual reproduction and plant infection.

### 3.3. Identification of Suppressor Mutations in FgSFL1

**Figure 3:**
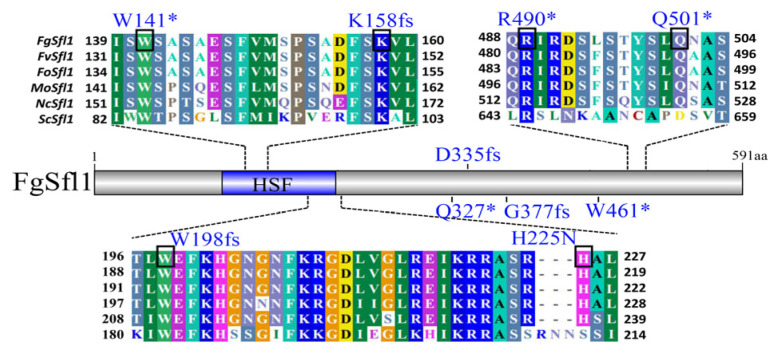
Schematic drawing of mutation sites identified in SFL1. Amino acid changes in *SFL1* identified in 25 suppressor strains are labeled above the schematic drawing of Sfl1 or sequence alignments of Sfl1 and its orthologs from *Fusarium* *oxysporum* (Fo), *Fusarium* *verticillioides* (Fv), *Magnaporthe* *oryzae* (Mo), *Neurospora* *crassa* (Nc), and *Saccharomyces* *cerevisiae* (Sc). The conserved amino acid residues with suppressor mutation in Sfl1 are labelled with black boxes. The predicted heat shock transcription factor binding domain (HSF) are labelled with the blue box. “fs”, frameshift variant; “*”, stop gained.

To identify suppressor mutations, we subjected the three suppressor strains HS-20, HS-21, and HS-25, which had disease indices of 59.0%, 57.4%, and 60.7% of the wild-type index, respectively (Table 2), to the wild-type strain PH-1 and *cpk1 cpk2* double mutant for whole genome sequencing (WGS). Interestingly, all three selected suppressor strains had mutations in *FgSFL1* (FGRRES_10868), which is orthologous to *SFL1* of *S. cerevisiae*. HS-25 had a non-sense mutation at Q501, and the two others had frameshift mutations at D335 and W198, respectively. We then sequenced the *FgSFL1* gene from the other 22 suppressor strains. All of them had mutations in *FgSFL1* at ten different sites (Figure 3). Thirteen suppressor strains had non-sense mutations at Q501, three suppressor strains had frameshift mutations at W198, and five suppressor strains had mutations in the HSF binding domain of FgSfl1 (Table 2). Only one suppressor strain had both a non-synonymous mutation at H225 and a non-sense mutation at R490.

### 3.4. Deletion of FgSFL1 Partially Rescued Defects of the cpk1 cpk2 Double Mutant

**Figure 4:**
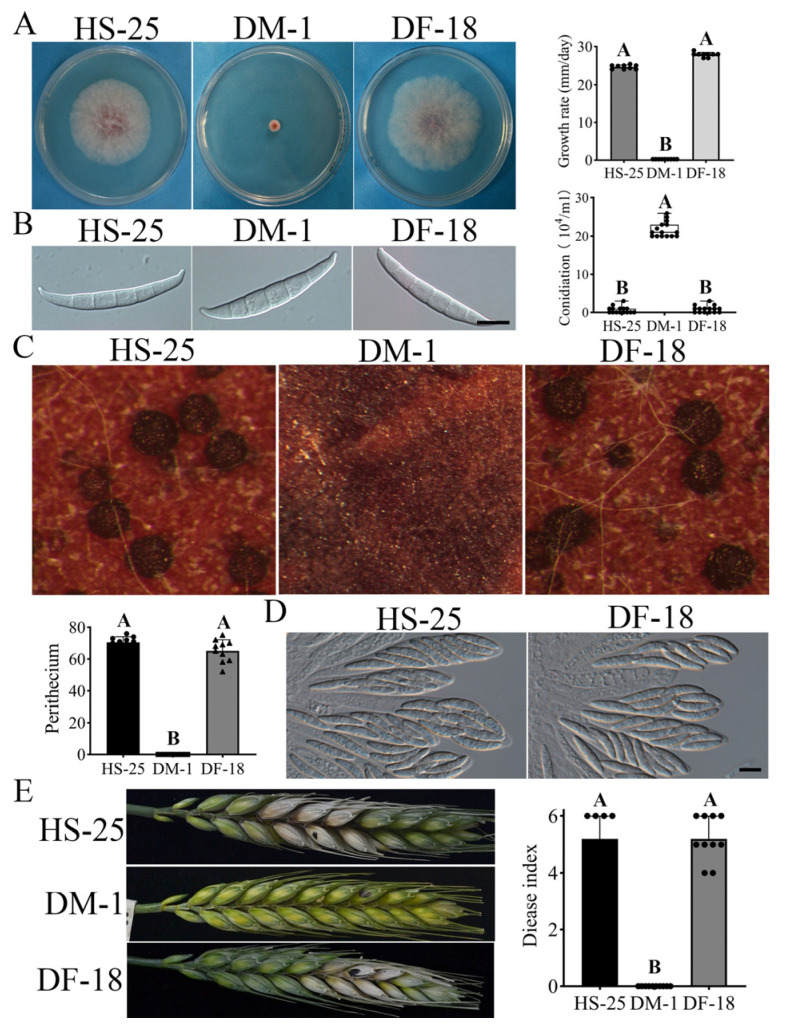
Verification of the suppressor mutations in FgSFL1. (**A**) Three-day-old PDA cultures of the wild-type strains PH-1, DM-1, and DF-18. (**B**) Conidiation of the wild-type strain PH-1, DM-1, and DF-18 after cultivation in CMC for five days. Bar = 20 µm. (**C**) Carrot agar cultures of PH-1, DM-1, and DF-18 were examined 7 days post-fertilization (dpf) and assay of perithecia of the wild-type strain PH-1, DM-1, and DF-18. (**D**) Ascospores of the wild-type strains PH-1, DM-1, and DF-18 were examined by differential interference contrast (DIC). Bar = 10 µm. (**E**) Flowering wheat heads were drop-inoculated with conidia of the same set of strains and photographed 14 days post-inoculation (dpi). Black dots mark the inoculated spikelets. Different letters indicate significant differences based on ANOVA analysis followed by Duncan’s multiple range test (*p* = 0.01) in A, B.

To verify the suppressor mutations in *FgSFL1*, the gene replacement construct was transformed into the *cpk1 cpk2* double mutant strain DM-1, and we conducted a PCR analysis to confirm that the transformant strains lacked the *FgSFL1* gene (Figure S3). A total of four transformants DF-16, DF-18, DF-23, and DF-25 (Table 1), with the same phenotype were obtained. When the DF-18 was incubated on the PDA medium for 3 d, the colony grew normal and was similar to the suppresser strain HS-25 (Table 3; Figure 4A). Although strain DF-18 was not restored in the reduction of conidiation in the *cpk1 cpk2* double mutant (Table 3), the conidium morphology was normal (Figure 4B).

In the sexual stage, the DF-18 produced fewer perithecia with mature ascospores (Figure 4C,D). However, the cirrhi were not detectable after 7 days post-fertilization (dpf) on the carrot agar plates (Figure 4C). In the wheat head infection assays, unlike the *cpk1 cpk2* double mutant, strain DF-18 caused extensive discoloration beyond the inoculation site at 14 dpi, and the disease index was approximately the same as the suppressor strains (Figure 4E; Table 3). Therefore, the deletion of *FgSFL1* is responsible for all the phenotypes observed in the suppressor strains.

### 3.5. FgSFL1 Is Important for Conidiation and Sexual Reproduction

*FgSFL1* is predicted to encode a 591-amino acid protein with the typical structure of a heat shock factor (HSF) DNA binding domain (Figure S4). To determine its function, we generated the *sfl1* gene replacement transformant in the wild-type strain PH-1 (Table 1). Five *sfl1* mutant strains SF-1, SF-6, SF-39, SF-40, and SF-47 were confirmed by Southern blot hybridization (Figure S5).

**Figure 5:**
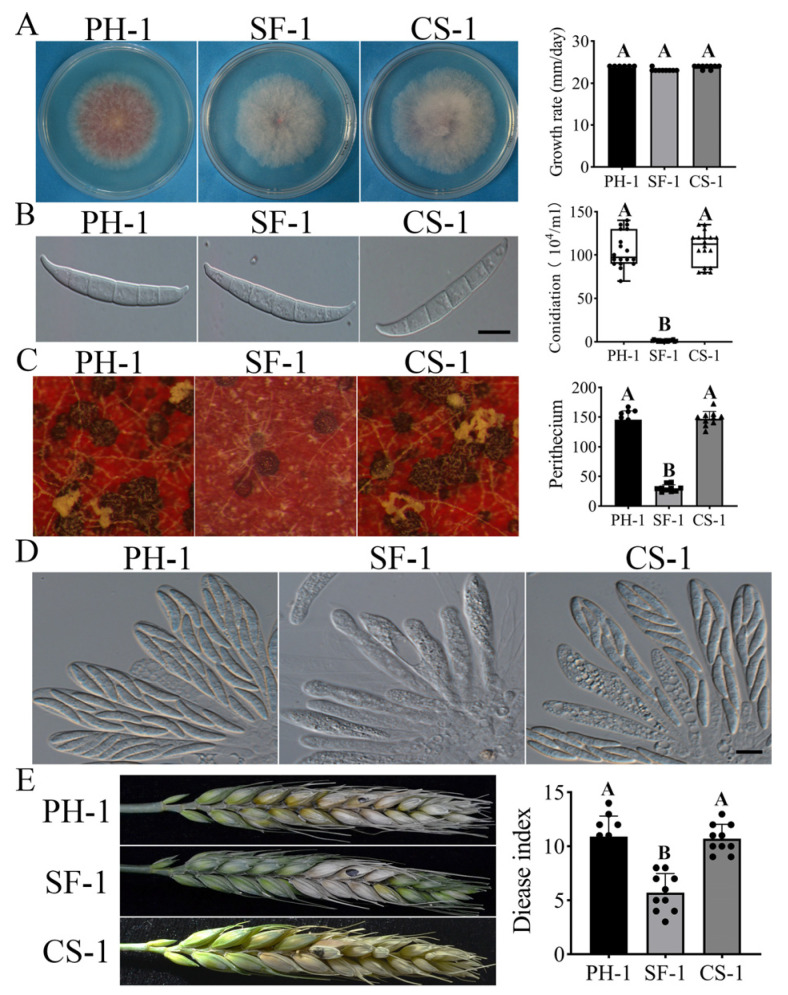
Defects of sfl1 in sexual reproduction and plant infection. (**A**) Three-day-old PDA cultures of the wild-type strains PH-1, SF-1, and CS-1. (**B**) Conidiation of the wild-type strains PH-1, SF-1, and CS-1 after cultivation in CMC for five days. Bar = 20 µm. (**C**) Carrot agar cultures of PH-1, SF-1, and CS-1 were examined 7 days post-fertilization (dpf) and assay of perithecia of the wild-type strains PH-1, SF-1, and CS-1. (**D**) Ascospores of the wild-type strains PH-1, SF-1, and CS-1 were examined by differential interference contrast (DIC). Bar = 10 µm. (**E**) Flowering wheat heads were drop-inoculated with conidia of the same set of strains and photographed 14 days post-inoculation (dpi). Black dots mark the inoculated spikelets. Different letters indicate significant differences based on ANOVA analysis followed by Duncan’s multiple range test (*p* = 0.01) in A, B.

The *sfl1* mutant strain SF-1 formed normal colonies similar to the wild-type on the PDA medium (Figure 5A), but were significantly reduced in conidiation (Table 3). In 5-day-old CMC cultures, the *Fgsfl1* deletion mutant produced less than 2% of the conidia than that of the wild-type strain PH-1 (Table 3). The conidia produced by SF-1 and the *Fgsfl1/FgSFL1* transformant CS-1 were normal in appearance, indistinguishable from the wild-type strain (Figure 5B).

On the self-mating carrot agar plates, the wild-type strain PH-1 and the CS-1 produced abundant perithecia and ascospore cirrhi after 7 dpf (Figure 5C). Under the same conditions, the *Fgsfl1* mutant produced only a few perithecia and no cirrhi (Figure 5C), and the ascospores did not mature properly (Figure 5D). After incubation for over 12 days, cirrhi were observed and the perithecia contained mature ascospores (Figure S2). Thus, losing the function of *FgSFL1* leads to a reduced yield of perithecia and delayed sexual development. These results indicate that the *FgSFL1* is important for conidiation and sexual development in *F. graminearum*.

### 3.6. FgSFL1 Is Also Important for Infection

In infection assays with flowering wheat heads, the wild-type strain PH-1 caused typical head blight symptoms and had a disease index of 12 ± 1.9 at 14 dpi. The average disease index of the SF-1 was 6 ± 2.2, reduced to almost 50% of the wild-type strain PH-1 (Figure 5E; Table 3). The CS-1 had a similar disease index as that of the wild-type strain PH-1 (Figure 5E; Table 3). These results show that the *FgSFL1* also plays an important role in plant infection in *F. graminearum*.

### 3.7. The T452D Mutation in Fgsfl1 Results in Reduced Growth

**Figure 6:**
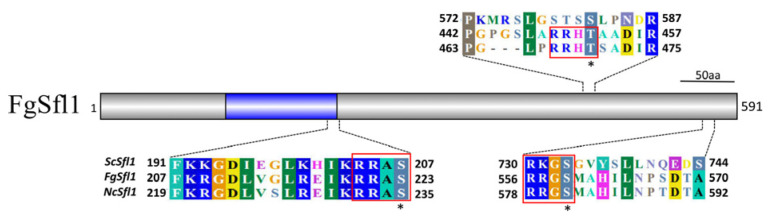
Site-directed mutagenesis of putative PKA phosphorylation sites in FgSfl1. Schematic drawing of the FgSfl1 protein and alignment of the marked region with its orthologs from *Neurospora crassa* (Nc) and *Saccharomyces cerevisiae* (Sc). The consensus PKA phosphorylation sites are enclosed with red lines. The putative PKA phosphorylation residues are marked with stars.

In *S. cerevisiae*, Sfl1 has two predicted consensus PKA phosphorylation sites, S207 and S733, that are conserved in FgSfl1 (S223 and S559). In addition, we found that T452 is another putative PKA phosphorylation site because it contains the [S/R] [S/R]-X-[S/T] consensus sequence (Figure 6) and is conserved in Fgsfl1 and its orthologs from other filamentous ascomycetes (Figure S4).

**Figure 7:**
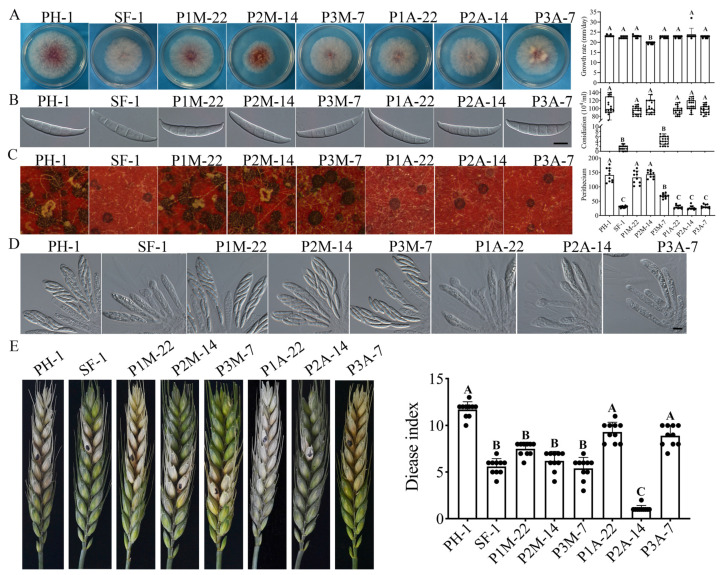
Phosphorylation site of *FgSFL1* is important in conidiation, sexual reproduction, and plant infection. (**A**) Three-day-old PDA cultures of the wild-type strains PH-1, P1M-22, P2M-14, P3M-7, P1A-22, P2A-14, and P3A-7. (**B**) Conidiation of the wild-type strains PH-1, P1M-22, P2M-14, P3M-7, P1A-22, P2A-14, and P3A-7 after cultivation in CMC for five days. Bar = 20 µm. (**C**) Carrot agar cultures of the wild-type strains PH-1, P1M-22, P2M-14, P3M-7, P1A-22, P2A-14, and P3A-7 were examined 7 days post-fertilization (dpf) and assay of perithecia of the wild-type strains PH-1, P1M-22, P2M-14, P3M-7, P1A-22, P2A-14, and P3A-7. (**D**) Ascospores of the wild-type strains PH-1, P1M-22, P2M-14, P3M-7, P1A-22, P2A-14, and P3A-7 were examined by differential interference contrast (DIC). Bar = 10 µm. (**E**) Flowering wheat heads were drop-inoculated with conidia of the same set of strains and photographed 14 days post-inoculation (dpi). Black dots mark the inoculated spikelets. Different letters indicate significant differences based on ANOVA analysis followed by Duncan’s multiple range test (*p* = 0.01) in A, B, C.

To determine the role of these three PKA phosphorylation sites, the putative *FgSFL1*^S223D^, *FgSFL1*^T452D^, and *FgSFL1*^S559D^ constitutive active alleles were generated and transformed into the *Fgsfl1* deletion mutant strain SF-1. As the control, we also generated the *FgSFL1*^S223A^, *FgSFL1*^T452A^, and *FgSFL1*^S559A^ alleles and transformed them into SF-1. In the resulting transformants of the *Fgsfl1* mutant that were verified for the expression of transforming mutant alleles of *FgSFL1*, only the *Fgsfl1/FgSFL1*^T452D^ transformant P2M-14 grew more slowly than the wild-type strain PH-1 (Figure 7A). Transformants expressing the other mutant alleles of *FgSFL1*, including *FgSFL1*^T452A^, were normal in growth (Figure 7A). Thus, phosphorylation at T452 negatively affects vegetative growth in *F. graminearum*.

### 3.8. The S559D Mutation in Fgsfl1 Affects Conidiation and Sexual Reproduction

Our analyses of conidiation indicated that only the *Fgsfl1/FgSFL1*^S559D^ transformant P3M-7, similar to SF-1, produced fewer than 4% of the conidia produced by the wild-type strain PH-1 (Table 3). All other strains were not significantly defective in conidiation (Table 3; Figure 7B). These results indicate that the S559 phosphorylation site of the FgSfl1 affects conidiation.

When assayed for sexual reproduction on carrot agar plates, the *Fgsfl1/FgSFL1*^S559D^ transformant P3M-7 produced fewer perithecia than the wild-type strain PH-1 but produced more perithecia than the *Fgsfl1* mutant (Figure 7C). After incubation for over 12 days, cirrhi were observed on perithecia (Figure S2).

### 3.9. The S-to-A Mutation at S223, T452, or S559 Affects Perithecium Development and Ascospore Release

Unlike transformant P3M-7, the *Fgsfl1/FgSFL1*^S223D^ and *Fgsfl1/FgSFL1*^T452D^ transformants were normal in perithecium formation and the production of ascospore cirrhi (Figure 7C). However, under the same conditions, transformants P1A-22, P2A-14, and P3A-7, expressing the FgSfl1 alleles with the S223A, T452A, and S559A mutations, respectively, produced fewer perithecia and had no visible ascospore cirrhi at 7 dpf (Figure 7C). Similar to the SF-1, after incubation for over 12 days, ascospore cirrhi with mature ascospores were observed in the perithecia (Figure S2). Although the P3M-7, P1A-22, P2A-14, and the P3A-7 were defective in perithecia production, their ascospores were similar to those of the wild-type strain PH-1 (Figure 7D). These results show that all three phosphorylation sites are important for the normal functions of FgSfl1 during sexual reproduction.

### 3.10. Phosphorylation Site in FgSfl1 Essential for Infection

Although the S559 phosphorylation site of FgSfl1 was responsible for conidiation and sexual reproduction and the T452 phosphorylation site of FgSfl1 was responsible for growth, all three of these phosphorylation site mutant strains showed defects in the infection of flowering wheat heads, except the P1A-22 and P3A-7. The average disease indices of the P1M-22, P2M-14, and P3M-7 were 8 ± 1.2, 7 ± 1.5, and 6 ± 2.1, respectively. All the disease indices of the three transformant strains were similar to the SF-1 (Figure 7E; Table 3). Interestingly, the P1A-22 and P3A-7 had almost no defects in the infection of flowering wheat heads (Figure 7E; Table 3). The P2A-14 caused typical symptoms only on the inoculated kernels, similar to that of the DM-1. These data indicate that all the phosphorylation sites of FgSfl1 are important for plant infection, and that the T452A and S559A phosphorylation sites of FgSfl1 may affect other processes in *F. graminearum*.

### 3.11. RNA-Seq Analysis of the Fgsfl1 Mutant

To identify genes affected by the deletion of *FgSFL1*, we performed an RNA-seq analysis with RNA isolated from hyphae at 5-dpf. In comparison with the wild-type strain PH-1, 263 genes were up-regulated 2-fold or more in the *Fgsfl1* deletion mutant (Table S2). Among the lower 49 genes with over 10-fold up-regulation, only one had ortholog to *S. cerevisiae* (Table S2) fatty acid synthase FAS1, and it was reported that the fatty acid’s metabolism is important to the virulence of *F. graminearum* [32]. The deletion of *FgSFL1* appeared to increase the expression of a number of genes related to infectivity, including one hsp70 protein (FGSG_06304), one hsp70 like protein (FGSG_06014), one G-protein coupled receptor protein (FGSG_04923), two glycotransferases, and three ion transporter proteins (Table S2). A gene ontology (GO) enrichment analysis showed that genes encoding membrane, ion transport, or amino acid transport were enriched in genes up-regulated in the *Fgsfl1* mutant (Figure S6). The up-regulation of these genes in the *Fgsfl1* mutant suggests that they may be important for infection.

A total of 337 genes were down-regulated in the *Fgsfl1* mutant compared to the wild type (Table S3). Of those, 32 were reduced over 10-fold reduction and most of them encoded hypothetical proteins. A GO analysis showed that the down-regulated genes were likely related to an oxidation-reduction process, oxidoreductase activity, transmembrane transport, and membrane (Figure S7). Many of these down-regulated genes may be related to the development of toxins in the wild type, including FGSG_01000 and FGSG_05839 that are homologous to yeast *ERG11* and *STL1* [33,34,35]. The deletion of *FgSFL1* also reduced the expression of several genes related to toxin synthesis, including the galactose oxidase (FGSG_11032), sarcosine oxidase (FGSG_02894), coenzyme a synthetase (FGSG_06462), and mannose-binding lectin (FGSG_11368). Reduction in the expression of these genes may be partly responsible for toxins produced in the *Fgsfl1* mutant.

## 4. Discussion

The cAMP-PKA signaling pathway is involved in many cellular progresses in various fungi, such as nutrient sensing, pseudohyphal differentiation, spore germination, and infection processes [10,36,37,38]. Like many other filamentous ascomycetes, *Fusarium graminearum* contains two genes, *FgCPK1* and *FgCPK2*, encoding the catalytic subunits of cAMP-dependent protein kinase A. Knocking out both *FgCPK1* and *FgCPK2* results in severe defects in growth, conidiation, sexual reproduction, and virulence [14]. In addition, the *cpk1 cpk2* double mutant was unstable and produced fast-growing spontaneous sectors in the original colony. Suppressor mutations partially recovered some of the defects in the *cpk1 cpk2* double mutant. In this study, we chose three suppressor strains with different disease indices for whole-genome sequencing [39] and found that the three selected suppressor strains had a mutation in *FgSFL1.* The other suppressor strains were detected by a PCR analysis, which indicated that all suppressor strains contained mutations in *FgSFL1*. In *Magnaporthe oryzae*, 18 of 20 suppressor strains contained mutations in *MoSFL1*, which also restores growth defects of the *cpkA cpk2* double mutant [17]. Thus, *SFL1* is important for regulating cellular processes via the cAMP-PKA signaling pathway.

In *Saccharomyces cerevisiae*, *SFL1* is required for normal cell-surface assembly during vegetative growth, and the deletion of *SFL1* enhances pseudohyphal and invasive growth [40,41]. In *M. oryzae*, although the growth rate of the *Mosfl1* mutant was similar to that of the wild-type strain Ku80, conidiation was reduced by approximately 70% [42]. In *Candida albicans*, the flocculation and filamentation is suppressed by Sfl1, and it acts as a novel negative regulator to control the morphogenesis of *C. albicans* [43]. We also found that the deletion of *SFL1* in *F. graminearum* negatively affected conidiation but not growth rate. In addition, the *sfl1* deletion mutant was defective in perithecia production and reduced by almost 50% in the ability to infect wheat heads, but DON production was not affected (Table S5). This suggests that *FgSFL1* does not regulate the expression of the *TRI* genes [44].

*Sfl1* is a downstream target of PKA in yeast and is a substrate of *Tpk2* [16]. Its function is controlled by PKA activity [45]. The S207 and S733 were the predicted PKA phosphorylation sites of *Sfl1* [46]. Because the *tpk1 tpk2 tpk3* triple mutant is non-viable, it is not clear whether the phosphorylation site of *Sfl1* could suppress the growth defect of the triple mutant. In *M. oryzae*, only the *MoSFL1*^S211D^ rescued the growth defect of the *cpkA cpk2* double mutant but not the appressorium formation [17]. In *F. graminearum*, the S223 and S559 phosphorylation sites were conserved and aligned with *S. cerevisiae* (Figure S4). We also found another potential phosphorylation site, T452, according to the features of the PKA phosphorylation site sequence that was also conserved in *M. oryzae*, *N. crassa*, *F. verticillioides*, and *F. oxysporum* (Figure S4). In this study, none of the constitutive active or inactive alleles could imitate the wild-type or *Fgsfl1* mutant in all phenotypes that were assayed, indicating that the three phosphorylation sites are precisely regulated by the PKA kinase during different development stages. All the transformant strains had normal growth rates except *Fgsfl1*/*FgSFL1*^S452D^ transformant P2M-22 (Table 3; Figure 7A). In *M. oryzae*, phosphorylated S211 disrupted its interaction with the Cyc8-Tup1 co-repressor, which prevented its ability to block the transcription of downstream genes that are important for growth [17]. In *F. graminearum*, the T452 to D mutation may have similar negative effects. In the virulence assay, the *Fgsfl1*/*FgSFL1*^S452A^ transformation strain had a similar disease index to the *cpk1 cpk2* double mutant (Table 3), indicating that phosphorylation of T452 by PKA is essential for pathogenesis. During conidial development, only the *Fgsfl1*/*FgSFL1*^S559D^ transformation strain was severely defective in conidiation (Table 3), suggesting that the non-phosphorylated S559 plays an important role in conidiation. In *M. oryzae*, the C-terminal region of MoSfl1 interacts with Cyc8 [17]. Thus, the S559 may also affect the interaction with Cyc8 and result in some defects in *F. graminearum*.

All three transformation strains with non-phosphorylated alleles had a similar sexual reproduction phenotype to the *Fgsfl1* mutant. Apparently, all three phosphorylation sites are essential for the function of *FgSFL1* in regulating sexual development. However, the S559 to D mutation also was defective in perithecia production and ascospore discharge (Figure 7C), indicating that, in the complex sexual reproduction process, the S559 phosphorylated state must be precisely controlled during different sexual stages. According to the above results, the functions of the phosphorylation sites are different between *F. graminearum* and *M. oryzae*. In *F. graminearum*, all the three phosphorylation sites are important, as they were responsible for growth, sexual reproduction, and infection progress though the precise regulation of phosphorylation.

All our collected suppressor strains have a mutation in the *FgSFL1*. In *M. oryzae*, mutations in *MoSFL1* also recovered growth defects of the *cpkA cpk2* double mutant [17]. It is likely that *SFL1* is prone to mutate in the *cpkA cpk2* double mutant, but *FgSFL1* was not located in the fast-speed sub-genome with a high frequency of variants [47]. Such a high probability of the mutation in the *SFL1* orthologs may indicate that *SFL1* plays a conserved role in cellular progresses in filamentous ascomycetes. According to the results of this study, the deletion of *FgSFL1* will significantly restore the growth rate of the *cpk1 cpk2* double mutant. Therefore, the suppressor strains with a mutation in *FgSFL1* are readily detected and recovered. This might obscure more significant mutational events, such as the mutation sites that can fully restore the defects in virulence or sexual development. The *cpk1 cpk2* double mutant is severely defective in various processes, such as penetration of wheat epidermis and sexual fruiting body production [14]. The spontaneous suppressor strains obtained during wheat inoculation or on the sexual induction medium may help identify more downstream genes involved in infection and sexual development regulated by the PKA pathway.

In summary, the *cpk1 cpk2* mutant was unstable and produced fast-growing sectors. All collected suppressor strains had a colony morphology comparable to the wild-type strain PH-1. They had been identified mutating in *FgSFL1* by a whole-genome sequence [39]. By knocking out the *FgSFL1* in the *cpk1 cpk2* double mutant, we identified the defects of the *cpk1 cpk2* double mutant that could be rescued by the loss of *FgSFL1*. In *F. graminearum*, *SFL1* is important for sexual development, conidiation, and infection. Furthermore, the S559 phosphorylation site is most important in repressing the function of *FgSfl1*.

## Figures and Tables

**Table 1:** Wild-type and mutant strains of *F. graminearum* used in this study.

Strains	Brief Description	Reference
PH-1	Wild-type	[18]
DM-1	*cpk1 cpk2* deletion mutant of PH-1	[14]
DF-18, DF-16, DF-23, DF-25	*Fgsfl1* deletion mutant of DM-1	This study
SF-1, SF-6, SF-39, SF-40, SF-47	*Fgsfl1* deletion mutant of PH-1	This study
CS-1	*Fgsfl1/FgSFL1* complemented transformant	This study
P1A-22, P1A-18, P1A-20	*FgSFL1*^S223A^ transformant of SF-1	This study
P1M-22, P1M-11, P1M-19	*FgSFL1*^S223D^ transformant of SF-1	This study
P2A-14, P2A-10, P2A-8	*FgSFL1*^T452A^ transformant of SF-1	This study
P2M-14, P2M-19, P2M-7	*FgSFL1*^T452D^ transformant of SF-1	This study
P3M-7, P3M-2, P3M-9	*FgSFL1*^S559S559D^ transformant of SF-1	This study
P3A-7, P3A-4, P3A-10	*FgSFL1*^S559S559A^ transformant of SF-1	This study
HS-1 to HS-25	Spontaneous suppressor of DM-1	This study

**Table 2:** Suppressor strains of the *cpk1 cpk2* double mutant and their growth rate, conidiation, wheat infection index, and various mutations in SFL1.

Strain	Growth Rate (%)	Conidiation (%)	Disease Index	Mutation in SFL1	
			(%)	Amino Acid	Nucleotide
PH-1	100	100	100	wild type	wild type
DM-1	NA	20.5	0	wild type	wild type
HS-1	95.4	1.5	16.4	K158fs	A^531^ to ΔA^531^
HS-2	95.4	2.8	18.9	Q501 *	C^1717^ to T^1717^
HS-3	100	2.8	25.4	Q501 *	C^1717^ to T^1717^
HS-4	95.8	1.7	38.5	Q501 *	C^1717^ to T^1717^
HS-5	90.8	2.0	2.5	Q501 *	C^1717^ to T^1717^
HS-6	92.3	1.0	20.5	Q501 *	C^1717^ to T^1717^
HS-7	104.9	3.0	6.6	W141 *	G^479^ to A^479^
HS-8	105.3	0.5	49.2	G377fs	Insertion of A at T^1347^
HS-9	102.3	0.7	49.2	W198fs	TG^759–760^ to ΔTG^759–760^
HS-10	102.2	0.4	21.3	Q327 *	C^1195^ to T^1195^
HS-11	100	0.5	4.9	Q327 *	C^1195^ to T^1195^
HS-12	100	1.5	26.2	Q501 *	C^1717^ to T^1717^
HS-13	100	1.4	49.2	W198fs	TG^759–760^ to ΔTG^759–760^
HS-14	95.4	1.2	31.1	141Trp(W) *	G^479^ to A^479^
HS-15	96.6	0.6	12.3	Q501 *	C^1717^ to T^1717^
HS-16	95.4	1.0	20.5	W461 *	G^1598^ to A^1598^
HS-17	95.4	1.2	4.1	Q501 *	C^1717^ to T^1717^
HS-18	100	2.3	23.0	Q501 *	C^1717^ to T^1717^
HS-19	95.9	0.4	36.1	Q501 *	C^1717^ to T^1717^
HS-20	98	0.7	59.0	D335fs	Insertion of A at T^1221^
HS-21	95	2.0	57.4	W198fs	TG^759–760^ to ΔTG^759–760^
HS-22	80.5	2.7	6.6	H225N; R490 *	C^840^ to A^840^; C^1684^ to T^1684^
HS-23	90.5	1.2	6.6	Q501 *	C^1717^ to T^1717^
HS-24	90.6	1.6	27.9	Q501 *	C^1717^ to T^1717^
HS-25	96	0.7	60.7	Q501 *	C^1717^ to T^1717^

fs: frameshift mutation; Δ: deletion mutation; *: termination mutation.

**Table 3:** Phenotypes of the *Fgsfl1* mutant in growth, conidiation, and plant infection.

Strain	Growth Rate (mm/Day) ^a,b^	Conidiation (×10^4^ Conidia/mL) ^a,c^	Disease Index ^a,d^
PH-1	23.4 ± 0.1 ^A^	105.8 ± 2.2 ^A^	12 ± 1.9 ^A^
SF-1	23.0 ± 0.1 ^A^	1.3 ± 0.3 ^B^	6 ± 2.2 ^B^
CS-1	23.3 ± 0.0 ^A^	108.3 ± 3.0 ^A^	10 ± 4.0 ^A^
DF-18	23.4 ± 0.2 ^A^	0.9 ± 2.1 ^B^	5 ± 1.6 ^A^
HS-20	23.1 ± 0.0 ^A^	0.8 ± 1.1 ^B^	6 ± 1.0 ^B^
HS-25	22.4 ± 0.6 ^A^	0.8 ± 0.9 ^B^	6 ± 0.57 ^B^
P1M-22	23.3 ± 0.1 ^A^	93.3 ± 2.9 ^A^	8 ± 1.2 ^B^
P2M-14	20.0 ± 0.2 ^B^	101.9 ± 1.3 ^A^	7 ± 1.5 ^B^
P3M-7	23.0± 0.1 ^A^	4.2 ± 1.7 ^B^	6 ± 2.1 ^B^
P1A-22	23.0 ± 0.1 ^A^	94.7 ± 4.2 ^A^	10 ± 1.7 ^A^
P2A-14	23.0 ± 0.2 ^A^	108.1 ± 4.6 ^A^	1 ± 2.4 ^C^
P3A-7	23.0 ± 0.2 ^A^	96.7 ± 1.4 ^A^	10 ± 1.9 ^A^

**^a^** Standard deviation (mean ± standard deviation) were calculated from at least three independent measurements; **^b^** average daily extension of colony radius; **^c^** conidiation was measured with 5-day-old Carboxymethylcellulose (CMC) culture; **^d^** diseased spikelets per wheat head examined 14 dpi. Mean and standard deviation were calculated with results from three replicates for growth rate and conidiation assays and ten replicates for disease index assays. Data were analysed with Duncan’s pairwise comparison. Different capital letters mark statistically significant differences (*p* = 0.01).

## Supplementary Materials

The following are available online at https://www.mdpi.com/article/10.3390/jof7090755/s1, Figure S1: Growth of spontaneous suppressor strains in race tube after cultivation for 15 days, Figure S2: Analysis of ascospores discharge from HS-20, HS-25, DF-18, SF-1, P1A-22, P2A-14, and P3A-7 on carrot plate for 12 days, Figure S3: Putative *sfl1 cpkA cpk2* mutants were screened by PCR analysis, Figure S4: Sequence alignment of *SFL1*, Figure S5: Putative *sfl1* mutants were screened by southern blot analysis, Figure S6: GO analysis of up-regulated genes in the 12-hours hyphae of the wild-type strain PH-1 and the *Fgsfl1* mutant, Figure S7: GO analysis of down-regulated genes in the 12-hours hyphae of the wild-type strain PH-1 and the *Fgsfl1* mutant, Figure S8: qRT-PCR analysis of the wild-type stain PH-1 and the *Fgsfl1* mutant, Table S1: PCR primers used in this study, Table S2: Genes up-regulated in the *Fgsfl1* mutant in comparison with the wild type, Table S3: Genes down-regulated in the *Fgsfl1* mutant in comparison with the wild type, Table S4: Analysis DON production in the *Fgsfl1* mutant.
