# Supplementary material for: FgSfl1 and Its Conserved PKA Phosphorylation Sites Are Important for Conidiation, Sexual Reproduction, and Pathogenesis in Fusarium graminearum
Source: J Fungi (Basel). 2021 Sep 14;7(9):755. doi: 10.3390/jof7090755 (PMC8466192; doi:10.3390/jof7090755)
Supplement: Supplementary file 1 [file jof-07-00755-s001.zip › new supplementary.pdf]

Journal of Fungi

MDPI

Supporting Information Legends

Table S1. PCR primers used in this study.

Name	Sequence(5'-3')
FgSFL1/1F	AACCGCTTGAACCAGCTACA
FgSFL1/2R（hph）	TTGACCTCCACTAGCTCCAGCCAAGCCAGATAAATGAAGGCAAGGTC
FgSFL1/2R（ble）	GTGTTGACCTCCACTAGCTCCAGCCACTGATGGTTAGTCGACAAGAAGGAT
FgSFL1/3F（hph）	GAATAGAGTAGATGCCGACCGCGGGTTGGCAGGCATTCTGTTTGACC
FgSFL1/3F（ble）	ACGAATTGCTTGCAGGCATCTCATGACGACGACAGAAAGAGAAAGC
FgSFL1/4R	ACCCTTGACTGGCACTGAAT
FgSFL1/5F	ATACAAAGCCTGTGACATCGC
FgSFL1/6R	AAGGGACATTCAGAGTAGGA
FgSFL1/7F	CTGTGCTGTTTCGTCAATC
FgSFL1/8R	CGACTCAAACCCAACTCAT
FgSFL1/CF	CGACTCACTATAGGGCGAATTGGGTACTCAAATTGGATTCCCGTCCTTCTTCCCACAC
FgSFL1/CR2	CACCACCCCGGTGAACAGCTCCTCGCCCTTGCTCACACCCTTGACTGGCACTGAAT
FgSFL1/R FLAG-HA	CCTCCTCGCCCTTGCTCACTTAATGTACCCATACGATGTTCCAGATTACGCTCTTGTCATCGTCATCCTTGTAATC
FgSFL1^{S223D}/F	TACGTGAGATCAAGCGTCGAGCCGATAGGCATGC
FgSFL1^{S223D}/R	CAAGTGCATGCCTATCGGCTCGACGCTTGATCTCACGTA
FgSFL1^{T452D} /F	CCTGGTAGCTTAGCACGCCGTCACGATGCTGCCGACATCC
FgSFL1^{T452D}/R	GGATGTCGGCAGCATCGTGACGGCGTGCTAAGCTACCAGG
FgSFL1^{S559D}/F	CCCACAACTCGACGTGGAGACATGGCTCATATTCTCAACCCGA
FgSFL1^{S559D}/R	ATGAGCCATGTCTCCACGTCGAGTTGTGG
FgSFL1^{S223A}/F	TACGTGAGATCAAGCGTCGAGCCGCTAGGCATGC
FgSFL1^{S223A}/R	CAAGTGCATGCCTAGCGGCTCGACGCTTGATCTCACGTA
FgSFL1^{T452A} /F	CCTGGTAGCTTAGCACGCCGTCACGCTGCTGCCGACATCC
FgSFL1^{T452A}/R	GGATGTCGGCAGCAGCGTGACGGCGTGCTAAGCTACCAGG
FgSFL1^{S559A}/F	CCCACAACTCGACGTGGAGCCATGGCTCATATTCTCAACCCGA
FgSFL1^{S559A}/R	ATGAGCCATGGCTCCACGTCGAGTTGTGG
YG/F	GATGTAGGAGGGCGTGGATATGTCCT
HY/R	GTATTGACCGATTCCTTGCGGTCCGAA
HYG/F	GGCTTGGCTGGAGCTAGTGGAGGTCAA
HYG/R	AACCCGCGGTCGGCATCTACTCTATTC
H852	ATGTTGGCGACCTCGTATTGG
H850	TTCCTCCCTTTATTTCAGATTCAA
H855/R	GCTGATCTGACCAGTTGC
H856/F	GTCGATGCGACGCAATCGT
BLE/F	CGAGCTCACTACACTAAACTCCCCGCTTCTCTC
BLE/R	TCATGAGATGCCTGCAAGCA
B850/F	TCAGCCCACTTGTAAGCAGTAGC
B852/R	CTGGATGCCGACGGATTTG
PFL2 insert/F	TAACGCCAGGGTTTTCCCAGTCA
PFL2 insert/R	CGTGCTGCTTCATGTGGTCGG
TUP1 Seq/F	AGAGGGTGTGCGAACAGAGT
TUP1 Seq/R	TCCTACCCATCCTTGACGGC
MSN2 Seq/F	TGCTAGTCCCTCTGTGGTG
MSN2 Seq/R	CTTCGCTACCTTGCCAGAC
RIM15 Seq/F	CCACTTGTGCTTCCGCTA
RIM15 Seq/R	CAATGCTGCTCGTGTGCCA
SOK1 Seq/F	AACCCACTCGCTTCATCG

SOK1 Seq/R	AATGTGAGTGTGAGTTCCG
YAK1 Seq/F	CAACGCCCGATAACCACGCA
YAK1 Seq/R	AGAATCCCAACTTCCAATCCA
CDTF1 Seq/F	TCAGAACTTAGTCCAACACACG
CDTF1 Seq/R	ACTCAGGACCTCATCGCCAC
SOM1 Seq/F	CTCGCGCCTTTTGGGATTAC
SOM1 Seq/R	CCGTGACAAATGATGCCTG
PDE2 Seq/F	ACCACCTCTTCAGACCCGAC
PDE2 Seq/R	ATTAGGGCGACTCAAAACC
01000F	GATCCTACGTTCCCATCATCTC
01000R	GATAGTGATCTCGGCCATCTTC
02139F	GCGAAGGAGCAATAGGGTTAT
02139R	ACGCTTTGAGTCTGGCTATG
02894F	ACTACTCCTACGAACCCTCTAC
02894R	TCTTGTCTGATGGACGAATCTG
04923F	CTACAGTGACGGCAGTCTTATC
04923R	CCAAGATAGCCAAACCAGTCT
05345F	CCCGAGTCAAGTCCTTCTTTG
05345R	GGCCAGTGACGTTCTCAATAA
05839F	GACCCTTTCCATGACTACTTCC
05839R	CAGATCACCGATCCAGAGAATG
06014F	GAAGAAGGTCAAGCGTCAATTC
06014R	GAAGCGTCCTCGGCTAATAC
06304F	TCAGATTGCCCACCGATTT
06304R	CTTGTATACCGGCTGCACATA
06462F	GTATCGCCGTCACAACATACT
06462R	TCCTCCCTCGTCGTCAATATAA
08264F	GTACTACCCACTGCGTCTTATT
08264R	GTGATGTTGTGGTGTTGGATG
08343F	GTCACTGGCTGTTGAGAAGTAT
08343R	CGAAAGACACTTTGGCACTTG
09821F	TCTCAAGAACGAAACCGTCAA
09821R	GGTGACTCAGGTCGAACATATC
11032F	GCGAAGTCTATAGCCCATCTTC
11032R	GTTGTCTGAACGGTACAATCCT
11368F	GTCTACCATGGCGAAGAGTG
11368R	TGACTAGGTTGCCATCTTCTTG
11656F	CTCGATCCAGATCAGCCATTAC
11656R	CATGGCAGAACGATCACTAAGA

Table S2. Genes up-regulated in the *Fgsfl1* mutant in comparison with the wild type.

Gene ID	MIPS ID	Broad ID	Gene Name[†]	Fold Change	Yeast_Top_Homolog (<1E-5)[‡]			NR Hits[§]
					Yeast_ID	Yeast_Gene	Yeast_Description	
FGRRES_16527	FGSG_05697	FGSG_05697		2.06				trehalose 6-phosphate synthase
FGRRES_04919	FGSG_04919	FGSG_04919		2.07				potassium sodium efflux p-type fungal-type
FGRRES_09053	FGSG_09053	FGSG_09053		2.07				uncharacterized protein y057_3636
FGRRES_13252_16931_M	FGSG_13252	FGSG_13252		2.07				uncharacterized protein y057_3069
FGRRES_09908	FGSG_09908	FGSG_09908	PKR1	2.07	YIL033C	BCY1	Regulatory subunit of the cyclic AMP-dependent protein kinase (PKA); PKA is a component of a signaling pathway that controls a variety of cellular processes, including metabolism, cell cycle, stress response, stationary phase, and sporulation	camp-dependent protein kinase regulatory subunit
FGRRES_07275	FGSG_07275	FGSG_07275		2.08				uncharacterized protein ffuj_03186
FGRRES_00172	FGSG_00172	FGSG_00172		2.08				glutathione s-transferase
FGRRES_07925	FGSG_07925	FGSG_07925		2.09				uncharacterized protein ffuj_13710
FGRRES_06190	FGSG_06190	FGSG_06190		2.14				plant basic secretory protein
FGRRES_12154	FGSG_12154	FGSG_12154		2.15	YIR038C	GTT1	ER associated glutathione S-transferase; capable of homodimerization;	glutathione s-transferase

				glutathione transferase for Yvc1p vacuolar cation channel; expression induced during the diauxic shift and throughout stationary phase; functional overlap with Gtt2p, Grx1p, and Grx2p	
FGRRES_05659	FGSG_05659	FGSG_05659	2.15		mfs phs inorganic phosphate transporter
FGRRES_02443_M	FGSG_02443	FGSG_02443	2.16		uncharacterized protein ffuj_07766
FGRRES_04639	FGSG_04639	FGSG_04639	2.18		uncharacterized protein ffuj_01997
FGRRES_03363	FGSG_03363	FGSG_03363	2.18		long-chain-fatty-acid-- ligase
FGRRES_16031	FGSG_02692	FGSG_02692	2.18		family taurine catabolism dioxygenase
FGRRES_07148	FGSG_07148	FGSG_07148	2.22		uncharacterized protein ffuj_02694
FGRRES_04913	FGSG_04913	FGSG_04913	2.24		related to beta-glucosidase
FGRRES_11924	FGSG_11924	FGSG_11924	2.24		hypothetical protein FGSG_11924 [Fusarium graminearum PH-1]
FGRRES_17323	FGSG_13710,FGSG_13711	FGSG_13710,FGSG_13711	2.24		uncharacterized protein lw93_13800
FGRRES_04182	FGSG_04182	FGSG_04182	2.24		carnitine transporter
FGRRES_03894_M	FGSG_03894	FGSG_03894	2.25		uncharacterized protein ffuj_14322
FGRRES_10843	FGSG_10843	FGSG_10843	2.29		histone-lysine n-methyltransferase ash1
FGRRES_00540	FGSG_00540	FGSG_00540	2.30		gnat family acetyltransferase
FGRRES_01631	FGSG_01631	FGSG_01631	2.32		integral membrane protein
FGRRES_15880	FGSG_01733	FGSG_01733	2.32		uncharacterized protein lw93_11253
FGRRES_03593	FGSG_03593	FGSG_03593	2.32		6-hydroxy-d-nicotine oxidase
FGRRES_10909	FGSG_10909	FGSG_10909	2.32		spfh domain band 7 family protein
FGRRES_08113_M	FGSG_08113	FGSG_08113	2.33		peptidase c14

FGRRES_05957	FGSG_05957	FGSG_05957	2.33				duf967 domain protein
FGRRES_00522	FGSG_00522	FGSG_00522	2.33				uncharacterized protein ffuj_01038
FGRRES_06188	FGSG_06188	FGSG_06188	2.35				uncharacterized protein ffuj_04959
FGRRES_10867	FGSG_10867	FGSG_10867	2.36				uncharacterized protein lw93_6273
FGRRES_02231	FGSG_02231	FGSG_02231	2.36	YAL049C	AIM2	Cytoplasmic protein involved in mitochondrial function or organization; null mutant displays reduced frequency of mitochondrial genome loss; potential Hsp82p interactor	dienelactone hydrolase
FGRRES_02924	FGSG_02924	FGSG_02924	2.36				sarcosine oxidase
FGRRES_02075	FGSG_02075	FGSG_02075	2.37				oxidoreductase
FGRRES_12760	FGSG_12760	FGSG_12760	2.37				uncharacterized protein ffuj_08719
FGRRES_11300	FGSG_11300	FGSG_11300	2.38				a retinal short-chain dehydrogenase reductase
FGRRES_04805	FGSG_04805	FGSG_04805	2.38				uncharacterized protein ffuj_02291
FGRRES_01751	FGSG_01751	FGSG_01751	2.40				serine threonine-protein kinase sgk2
FGRRES_13718	FGSG_13718	FGSG_13718	2.40				uncharacterized protein ffuj_07258
FGRRES_16137	FGSG_04191	FGSG_04191	2.41				regulatory protein for the arginine catabolic pathway
FGRRES_08023	FGSG_08023	FGSG_08023	2.41				isotrichodermin c-15 hydroxylase
FGRRES_03365	FGSG_03365	FGSG_03365	2.43				endonuclease exonuclease phosphatase family protein
FGRRES_07152	FGSG_07152	FGSG_07152	2.43				glucose repressible protein grg1
FGRRES_07896	FGSG_07896	FGSG_07896	2.45	YLL063C	AYT1	Acetyltransferase; catalyzes trichothecene 3-O-acetylation, suggesting a	trichothecene 3-o- partial

						possible role in trichothecene biosynthesis	
FGRRES_03288	FGSG_03288	FGSG_03288	2.46				acid beta-fructofuranosidase precursor
FGRRES_09502	FGSG_09502	FGSG_09502	2.48				uncharacterized protein ffuj_04369
FGRRES_12829	FGSG_12829	FGSG_12829	2.48				uncharacterized protein lw93_8250
FGRRES_10537	FGSG_10537	FGSG_10537	2.50				d-amino acid oxidase
FGRRES_16170	FGSG_03995	FGSG_03995	2.52				vegetative incompatibility protein het-e-1
FGRRES_16274_M	FGSG_03415	FGSG_03415	2.52				c6 transcription factor
FGRRES_17037	FGSG_08437	FGSG_08437	2.55				uncharacterized protein ffuj_06074
FGRRES_07829	FGSG_07829	FGSG_07829	2.56				extracellular serine-rich protein
FGRRES_04223	FGSG_04223	FGSG_04223	2.57				alcohol dehydrogenase (nadp+)
FGRRES_02557_M	FGSG_02557	FGSG_02557	2.57				vegetative incompatibility protein het-e-1
FGRRES_01532	FGSG_01532	FGSG_01532	2.59				uncharacterized protein lw93_11484
FGRRES_01650_M	FGSG_01650	FGSG_01650	2.59				protein png1
FGRRES_03785_M	FGSG_03785	FGSG_03785	2.59				triacylglycerol lipase
FGRRES_12418	FGSG_12418	FGSG_12418	2.61				uncharacterized protein lw93_10957
FGRRES_10162	FGSG_10162	FGSG_10162	2.63				uncharacterized protein y057_2444
FGRRES_10132	FGSG_10132	FGSG_10132	2.64				gtp-binding protein
FGRRES_01286	FGSG_01286	FGSG_01286	2.65				uncharacterized protein ffuj_01482
FGRRES_17643	FGSG_05066	FGSG_05066	2.67	YGR157W	CHO2	Phosphatidylethanolamine methyltransferase (PEMT); catalyzes the first step in the conversion of phosphatidylethanolamine to phosphatidylcholine during the methylation pathway of phosphatidylcholine biosynthesis	phosphatidylethanolamine n-methyltransferase

FGRRES_03395	FGSG_03395	FGSG_03395	2.68				uncharacterized protein lw94_14401
FGRRES_03500_M	FGSG_03500	FGSG_03500	2.69				uncharacterized protein lw93_3111
FGRRES_05834_M	FGSG_05834	FGSG_05834	2.69				uncharacterized protein lw94_11295
FGRRES_04525	FGSG_04525	FGSG_04525	2.70				p-loop containing nucleoside triphosphate hydrolase
FGRRES_15691	FGSG_00123	FGSG_00123	2.71				ankyrin repeat protein
FGRRES_08263	FGSG_08263	FGSG_08263	2.72				domain protein
FGRRES_02556	FGSG_02556	FGSG_02556	2.72				uncharacterized protein y057_2886
FGRRES_03163	FGSG_03163	FGSG_03163	2.73				flavin-binding monooxygenase
FGRRES_16357	FGSG_02984	FGSG_02984	2.76				phosphoethanolamine n-methyltransferase 3
FGRRES_13367	FGSG_13367	FGSG_13367	2.76				uncharacterized protein ffuj_06075
FGRRES_07847	FGSG_07847	FGSG_07847	2.78				group protein
FGRRES_04640	FGSG_04640	FGSG_04640	2.79				hsp70 protein
FGRRES_10728	FGSG_10728	FGSG_10728	2.79	YGR225W	AMA1	ctivator of meiotic anaphase promoting complex (APC/C); Cdc20p family member; required for initiation of spore wall assembly; required for Clb1p degradation during meiosis; prevents premature assembly of the meiosis I spindle, required for DSB induced prophase I arrest	fzr protein
FGRRES_00220	FGSG_00220	FGSG_00220	2.79				sodium-dependent serotonin transporter
FGRRES_17243	FGSG_09605	FGSG_09605	2.84				uncharacterized protein lw93_14006

FGRRES_09175	FGSG_09175	FGSG_09175	2.84	hypothetical protein FGSG_09175 [Fusarium graminearum PH-1]
FGRRES_03883	FGSG_03883	FGSG_03883	2.85	uncharacterized protein ffuj_11288
FGRRES_17678	FGSG_05820	FGSG_05820	2.85	related to methyltransferase
FGRRES_03822	FGSG_03822	FGSG_03822	2.87	uncharacterized protein lw93_6876
FGRRES_07596	FGSG_07596	FGSG_07596	2.89	uncharacterized protein y057_4894
FGRRES_09082	FGSG_09082	FGSG_09082	2.89	glucan -alpha-glucosidase
FGRRES_17502_M	FGSG_13897	FGSG_13897	2.90	amino-acid permease inda1
FGRRES_11324	FGSG_11324	FGSG_11324	2.91	ring finger protein 43
FGRRES_10154	FGSG_10154	FGSG_10154	2.91	uncharacterized protein lw93_13847
FGRRES_03072	FGSG_03072	FGSG_03072	2.91	tripeptidyl-peptidase i
FGRRES_12503	FGSG_12503	FGSG_12503	2.95	uncharacterized protein y057_5082
FGRRES_17585	FGSG_11245	FGSG_11245	3.00	uncharacterized protein ffuj_14339
FGRRES_08925	FGSG_08925	FGSG_08925	3.01	f-box domain-containing protein
FGRRES_05945	FGSG_05945	FGSG_05945	3.03	hypothetical protein FGSG_05945 [Fusarium graminearum PH-1]
FGRRES_15690	FGSG_00121	FGSG_00121	3.10	intracellular serine protease
FGRRES_00122	FGSG_00122	FGSG_00122	3.10	intracellular serine protease
FGRRES_04803	FGSG_04803	FGSG_04803	3.10	c6 transcription factor
FGRRES_11233	FGSG_11233	FGSG_11233	3.10	uncharacterized protein ffuj_14866
FGRRES_07448	FGSG_07448	FGSG_07448	3.14	uncharacterized protein y057_11785
FGRRES_06192	FGSG_06192	FGSG_06192	3.14	uncharacterized protein lw93_10823
FGRRES_12132	FGSG_12132	FGSG_12132	3.18	serine threonine protein kinase
FGRRES_07734	FGSG_07734	FGSG_07734	3.19	hypothetical protein FGSG_07734 [Fusarium graminearum PH-1]
FGRRES_16623	FGSG_06238	FGSG_06238	3.20	chitinase
FGRRES_06199	FGSG_06199	FGSG_06199	3.22	trans-aconitate 2-methyltransferase
FGRRES_11509	FGSG_11509	FGSG_11509	3.23	uncharacterized protein lw93_10044

FGRRES_15993	FGSG_02430	FGSG_02430	3.23	uncharacterized protein ffuj_14028
FGRRES_16887	FGSG_07735	FGSG_07735	3.27	nacht domain-containing protein
FGRRES_09727	FGSG_09727	FGSG_09727	3.27	hypothetical protein FGSG_09727 [Fusarium graminearum PH-1]
FGRRES_13902	FGSG_13902	FGSG_13902	3.28	hypothetical protein FGSG_13902 [Fusarium graminearum PH-1]
FGRRES_15936	FGSG_02129	FGSG_02129	3.39	nacht domain protein
FGRRES_11190	FGSG_11190	FGSG_11190	3.39	guanyl-specific ribonuclease f1
FGRRES_02130	FGSG_02130	FGSG_02130	3.40	nfx1-type zinc finger-containing protein 1
FGRRES_02266	FGSG_02266	FGSG_02266	3.41	short-chain alcohol dehydrogenase
FGRRES_13083	FGSG_13083	FGSG_13083	3.42	hypothetical protein FGSG_13083 [Fusarium graminearum PH-1]
FGRRES_08196	FGSG_08196	FGSG_08196	3.45	peptidase a4 family protein
FGRRES_11103	FGSG_11103	FGSG_11103	3.45	aat family amino acid transporter
FGRRES_16905	FGSG_07817	FGSG_07817	3.45	uncharacterized protein ffuj_11405
FGRRES_00799	FGSG_00799	FGSG_00799	3.49	structure-specific endonuclease subunit slx4
FGRRES_15987	FGSG_02410	FGSG_02410	3.53	uncharacterized protein lw93_14927
FGRRES_16275	FGSG_03407	FGSG_03407	3.55	neutral amino acid permease protein
FGRRES_04490	FGSG_04490	FGSG_04490	3.56	uncharacterized protein ffuj_03572
FGRRES_17089	FGSG_08170	FGSG_08170	3.61	d-amino acid oxidase
FGRRES_06303	FGSG_06303	FGSG_06303	3.66	uncharacterized protein ffuj_13540
FGRRES_09501	FGSG_09501	FGSG_09501	3.74	uncharacterized protein lw93_14115
FGRRES_15856	FGSG_01507	FGSG_01507	3.78	uncharacterized protein lw93_11529
FGRRES_11105	FGSG_11105	FGSG_11105	3.80	heat shock 70 kda protein 12b
FGRRES_02131	FGSG_02131	FGSG_02131	3.82	uncharacterized protein lw94_1599
FGRRES_07772	FGSG_07772	FGSG_07772	3.82	hypothetical protein FGSG_07772 [Fusarium graminearum PH-1]

FGRRES_13849	FGSG_13849	FGSG_13849	3.83	uncharacterized protein ffuj_08417
FGRRES_03340	FGSG_03340	FGSG_03340	3.83	polyketide synthase
FGRRES_09412	FGSG_09412	FGSG_09412	3.84	uncharacterized protein ffuj_04249
FGRRES_17477	FGSG_11102	FGSG_11102	3.86	hypothetical protein FG05_11102 [Fusarium graminearum]
FGRRES_02354	FGSG_02354	FGSG_02354	3.87	chitinase
FGRRES_17306	FGSG_10047	FGSG_10047	3.91	uncharacterized protein ffuj_07399
FGRRES_07543	FGSG_07543	FGSG_07543	3.92	sugar transport protein stp1
FGRRES_11992	FGSG_11992	FGSG_11992	3.93	udpglucose 4-epimerase
FGRRES_09821	FGSG_09821	FGSG_09821	3.98	glycoside hydrolase family 43
FGRRES_07497	FGSG_07497	FGSG_07497	4.03	uncharacterized protein lw93_7934
FGRRES_00313	FGSG_00313	FGSG_00313	4.05	upc2-regulatory protein involved in control of sterol uptake
FGRRES_13203	FGSG_13203	FGSG_13203	4.08	regulator of nonsense transcripts 1 like protein
FGRRES_13573	FGSG_13573	FGSG_13573	4.08	hypothetical protein FGSG_13573 [Fusarium graminearum PH-1]
FGRRES_13917	FGSG_13917	FGSG_13917	4.11	hypothetical protein FGSG_13917 [Fusarium graminearum PH-1]
FGRRES_04870	FGSG_04870	FGSG_04870	4.11	uncharacterized protein ffuj_08255
FGRRES_15901	FGSG_01869	FGSG_01869	4.25	uncharacterized protein lw93_5916
FGRRES_07757	FGSG_07757	FGSG_07757	4.25	integral membrane protein pth11
FGRRES_07843	FGSG_07843	FGSG_07843	4.26	uncharacterized protein ffuj_12402
FGRRES_13987_M	FGSG_13987	FGSG_13987	4.28	myosin light chain smooth muscle
FGRRES_07899	FGSG_07899	FGSG_07899	4.28	uncharacterized protein ffuj_13596
FGRRES_02074	FGSG_02074	FGSG_02074	4.31	hypothetical protein FGSG_02074 [Fusarium graminearum PH-1]
FGRRES_16195	FGSG_03821	FGSG_03821	4.32	mrna 3 -end-processing protein yth1

FGRRES_11299	FGSG_11299	FGSG_11299	4.36				sterigmatocystin biosynthesis lipase esterase stci
FGRRES_08156	FGSG_08156	FGSG_08156	4.38				integral membrane protein
FGRRES_00046	FGSG_00046	FGSG_00046	4.39				multidrug resistance protein
FGRRES_06098_M	FGSG_06098	FGSG_06098	4.39	YOL058W	ARG1	Arginosuccinate synthetase; catalyzes the formation of L-argininosuccinate from citrulline and L-aspartate in the arginine biosynthesis pathway; potential Cdc28p substrate	argininosuccinate synthase
FGRRES_02285	FGSG_02285	FGSG_02285	4.40				isoflavone reductase like protein
FGRRES_09752	FGSG_09752	FGSG_09752	4.42				uncharacterized protein lw93_10612
FGRRES_07386	FGSG_07386	FGSG_07386	4.44				uncharacterized protein ffuj_02527
FGRRES_07894	FGSG_07894	FGSG_07894	4.46				phosphate:h+ symporter
FGRRES_13289	FGSG_13289	FGSG_13289	4.46				hypothetical protein FGSG_13289 [Fusarium graminearum PH-1]
FGRRES_04512	FGSG_04512	FGSG_04512	4.52				sodium potassium-transporting atpase subunit alpha
FGRRES_02139	FGSG_02139	FGSG_02139	4.55				canalicular multispecific organic anion transporter 2
FGRRES_08264	FGSG_08264	FGSG_08264	4.62				mg2+ transporter zinc transport protein
FGRRES_16560	FGSG_05866	FGSG_05866	4.63				serine threonine protein kinase
FGRRES_09827_M	FGSG_09827	FGSG_09827	4.65				uncharacterized protein ffuj_04670
FGRRES_02414	FGSG_02414	FGSG_02414	4.67				uncharacterized protein ffuj_00134
FGRRES_00036	FGSG_00036	FGSG_00036	4.71	YPL231W	FAS2	Alpha subunit of fatty acid synthetase; complex catalyzes the synthesis of long-	acyl transferase acyl hydrolase lysophospholipase

				chain saturated fatty acids; contains the acyl-carrier protein domain and beta-ketoacyl reductase, beta-ketoacyl synthase and self-pantetheinylation activities	
FGRRES_13901	FGSG_13901	FGSG_13901	4.73		hypothetical protein FGSG_13901 [Fusarium graminearum PH-1]
FGRRES_08616	FGSG_08616	FGSG_08616	4.75		hypothetical protein FGSG_08616 [Fusarium graminearum PH-1]
FGRRES_11993	FGSG_11993	FGSG_11993	4.82		uncharacterized protein y057_14791
FGRRES_03165	FGSG_03165	FGSG_03165	4.95		ethanolamine utilization protein
FGRRES_07844	FGSG_07844	FGSG_07844	5.05		hypothetical protein FGSG_07844 [Fusarium graminearum PH-1]
FGRRES_03834	FGSG_03834	FGSG_03834	5.12		low-affinity potassium transport protein
FGRRES_17372	FGSG_10471	FGSG_10471	5.19		helicase-like transcription factor protein
FGRRES_16893	FGSG_07776	FGSG_07776	5.21		arsenite efflux transporter -like
FGRRES_12570	FGSG_12570	FGSG_12570	5.33		gnat family protein
FGRRES_11298	FGSG_11298	FGSG_11298	5.34		fad-containing monooxygenase
FGRRES_11371	FGSG_11371	FGSG_11371	5.44		hypothetical protein FGSG_11371 [Fusarium graminearum PH-1]
FGRRES_04766	FGSG_04766	FGSG_04766	5.45		hypothetical protein FGSG_04766 [Fusarium graminearum PH-1]
FGRRES_15807	FGSG_00963	FGSG_00963	5.48		uncharacterized protein ffuj_00641
FGRRES_03414	FGSG_03414	FGSG_03414	5.53		dimethylglycine mitochondrial precursor
FGRRES_16588	FGSG_06013	FGSG_06013	5.54		ring finger domain-containing protein
FGRRES_13977	FGSG_13977	FGSG_13977	5.57		hypothetical protein FGSG_13977 [Fusarium graminearum PH-1]

FGRRES_11104	FGSG_11104	FGSG_11104	5.69	triacylglycerol lipase ii precursor
FGRRES_11362	FGSG_11362	FGSG_11362	5.74	uncharacterized protein ffuj_00281
FGRRES_04596	FGSG_04596	FGSG_04596	5.79	o-methyl transferase partial
FGRRES_04519	FGSG_04519	FGSG_04519	5.85	uncharacterized protein lw93_3464
FGRRES_03859	FGSG_03859	FGSG_03859	5.87	hypothetical protein FGSG_03859 [Fusarium graminearum PH-1]
FGRRES_16194	FGSG_03823	FGSG_03823	5.90	integral membrane protein
FGRRES_10143	FGSG_10143	FGSG_10143	5.96	hexamer-binding protein hexbp
FGRRES_16894	FGSG_07777	FGSG_07777	5.99	uncharacterized protein ffuj_14757
FGRRES_06015	FGSG_06015	FGSG_06015	6.20	hypothetical protein FGSG_06015 [Fusarium graminearum PH-1]
FGRRES_07813	FGSG_07813	FGSG_07813	6.30	hypothetical protein FGSG_07813 [Fusarium graminearum PH-1]
FGRRES_03416_M	FGSG_03416	FGSG_03416	6.40	fad dependent oxidoreductase
FGRRES_09268	FGSG_09268	FGSG_09268	6.55	uncharacterized protein ffuj_04143
FGRRES_12977	FGSG_12977	FGSG_12977	6.59	mfs monocarboxylate transporter
FGRRES_16073	FGSG_11631	FGSG_11631	6.63	mfs allantoate transporter
FGRRES_16631	FGSG_06304	FGSG_06304	6.65	hsp70 protein
FGRRES_04923	FGSG_04923	FGSG_04923	7.16	g-protein coupled receptor protein
FGRRES_05620	FGSG_05620	FGSG_05620	7.21	upf0145 domain-containing protein
FGRRES_16300_M	FGSG_12474,FGSG_12475	FGSG_12474,FGSG_12475	7.39	integral membrane protein
FGRRES_03972	FGSG_03972	FGSG_03972	7.51	isoamyl alcohol oxidase
FGRRES_04798	FGSG_04798	FGSG_04798	7.62	uncharacterized protein ffuj_11304
FGRRES_06014	FGSG_06014	FGSG_06014	7.68	hsp70-like protein
FGRRES_07997	FGSG_07997	FGSG_07997	7.86	uncharacterized protein lw94_10223
FGRRES_16108	FGSG_04466	FGSG_04466	7.98	ankyrin repeat protein
FGRRES_16012	FGSG_02548	FGSG_02548	8.24	uncharacterized protein y057_4303

FGRRES_16930	FGSG_07934	FGSG_07934	8.34	uncharacterized protein y057_3068
FGRRES_03460	FGSG_03460	FGSG_03460	8.52	peptide transporter
FGRRES_04871	FGSG_04871	FGSG_04871	8.79	conserved plasmodium protein
FGRRES_04689_M	FGSG_04690	FGSG_04690	8.80	uncharacterized protein lw93_10970
FGRRES_08343	FGSG_08343	FGSG_08343	9.34	h+-transporting atpase
FGRRES_10470	FGSG_10470	FGSG_10470	9.47	early growth response protein 1-b
FGRRES_08139	FGSG_08139	FGSG_08139	9.50	hypothetical protein FGSG_08139 [Fusarium graminearum PH-1]
FGRRES_00348	FGSG_00348	FGSG_00348	9.60	argonaute like protein
FGRRES_17094	FGSG_08151	FGSG_08151	9.70	feebly like protein
FGRRES_17387	FGSG_10548	FGSG_10548	9.72	polyketide synthase
FGRRES_16372	FGSG_02897	FGSG_02897	9.80	unnamed protein product [Fusarium graminearum]
FGRRES_01559	FGSG_01559	FGSG_01559	10.24	serine threonine protein kinase
FGRRES_15837	FGSG_11919,FGSG_11920	FGSG_11919,FGSG_11920	10.34	ankyrin repeat and ibr domain-containing protein 1
FGRRES_02318	FGSG_02318	FGSG_02318	10.48	kinesin light chain
FGRRES_07973	FGSG_07973	FGSG_07973	10.54	uncharacterized protein y057_6743
FGRRES_09224	FGSG_09224	FGSG_09224	10.74	uncharacterized protein ffuj_04185
FGRRES_16678	FGSG_06559	FGSG_06559	10.95	uncharacterized protein lw93_14219
FGRRES_01780	FGSG_01780	FGSG_01780	10.99	hypothetical protein FGSG_01780 [Fusarium graminearum PH-1]
FGRRES_16903	FGSG_07814	FGSG_07814	11.15	high-affinity methionine permease
FGRRES_09151_M	FGSG_09151	FGSG_09151	11.85	mg2+ transporter -like zinc transport protein
FGRRES_05345	FGSG_05345	FGSG_05345	11.86	
FGRRES_13525	FGSG_13525	FGSG_13525	11.93	uncharacterized protein ffuj_03966

FGRRES_17479	FGSG_11096	FGSG_11096	12.27				unnamed protein product [Fusarium graminearum]
FGRRES_04646_M	FGSG_04646	FGSG_04646	12.65				unnamed protein product [Fusarium graminearum]
FGRRES_09095	FGSG_09095	FGSG_09095	13.04				uncharacterized protein lw93_3439
FGRRES_04175	FGSG_04175	FGSG_04175	13.49				uncharacterized protein ffuj_09621
FGRRES_09441	FGSG_09441	FGSG_09441	13.55				uncharacterized protein lw93_14176
FGRRES_11656	FGSG_11656	FGSG_11656	13.67	YKL182W	FAS1	Beta subunit of fatty acid synthetase; complex catalyzes the synthesis of long-chain saturated fatty acids; contains acetyltransacylase, dehydratase, enoyl reductase, malonyl transacylase, and palmitoyl transacylase activities	fatty acid synthase subunit fungi type
FGRRES_12078	FGSG_12078	FGSG_12078	13.76				litaf-like zinc finger domain-containing protein
FGRRES_12902_M	FGSG_12902	FGSG_12902	14.13				uncharacterized protein ffuj_13539
FGRRES_04174	FGSG_04174	FGSG_04174	17.19				uncharacterized protein lw93_13222
FGRRES_06728	FGSG_06728	FGSG_06728	17.94				uncharacterized protein ffuj_03255
FGRRES_08246_M	FGSG_08246	FGSG_08246	18.88				zinc c2h2-type dna-binding partial
FGRRES_04465_M	FGSG_04465	FGSG_04465	18.97				sugar transporter
FGRRES_01705	FGSG_01705	FGSG_01705	19.17				flotillin
FGRRES_13411	FGSG_13411	FGSG_13411	19.76				hypothetical protein FGSG_13411 [Fusarium graminearum PH-1]
FGRRES_03825	FGSG_03825	FGSG_03825	19.81				large-conductance mechanosensitive channel

FGRRES_20172	FGSG_12438	FGSG_12438	20.45	cytochrome p450 oxidoreductase
FGRRES_00351_M	FGSG_00351	FGSG_00351	22.28	uncharacterized protein lw94_2250
FGRRES_09463	FGSG_09463	FGSG_09463	23.40	rtm1-like protein
FGRRES_04802	FGSG_04802	FGSG_04802	23.52	heterokaryon incompatibility protein
FGRRES_07496_M	FGSG_07496	FGSG_07496	23.58	high-affinity methionine permease
FGRRES_10542	FGSG_10542	FGSG_10542	26.01	uncharacterized protein ffuj_09281
FGRRES_10350	FGSG_10350	FGSG_10350	27.57	uncharacterized protein ffuj_07096
FGRRES_01752	FGSG_01752	FGSG_01752	27.93	hypothetical protein FGSG_01752 [Fusarium graminearum PH-1]
FGRRES_00049	FGSG_00049	FGSG_00049	30.13	branched-chain amino acid aminotransfer-ase
FGRRES_03197	FGSG_03197	FGSG_03197	31.35	uncharacterized protein lw94_8739
FGRRES_17032	FGSG_08450	FGSG_08450	31.92	uncharacterized protein ffuj_06061
FGRRES_16234	FGSG_03608	FGSG_03608	33.20	pathogenicity protein
FGRRES_00258	FGSG_00258	FGSG_00258	33.58	uncharacterized protein lw93_2879
FGRRES_13756	FGSG_13756	FGSG_13756	34.40	hydrolase or acyltransferase (alpha beta hydrolase superfamily)
FGRRES_16310	FGSG_03198	FGSG_03198	34.90	uncharacterized protein lw93_4980
FGRRES_03516	FGSG_03516	FGSG_03516	35.17	neutral amino acid permease
FGRRES_02468_M	FGSG_02468	FGSG_02468	37.73	uncharacterized protein ffuj_07794
FGRRES_17165	FGSG_09152	FGSG_09152	38.35	amino acid transporter
FGRRES_00312	FGSG_00312	FGSG_00312	62.59	uncharacterized protein ffuj_00814
FGRRES_08169	FGSG_08169	FGSG_08169	67.00	high-affinity methionine permease
FGRRES_12209_M	FGSG_12209	FGSG_12209	69.33	hypothetical protein FG05_12209 [Fusarium graminearum]
FGRRES_16935	FGSG_07968	FGSG_07968	102.42	related to rtm1p
FGRRES_05521	FGSG_05521	FGSG_05521	336.16	uncharacterized protein ffuj_08656

Table S3. Genes down-regulated in the Fgsfl1 mutant in comparison with the wild type.

Gene ID	MIPS ID	Broad ID	Gene Name[†]	Fold Change	Yeast_Top_Homolog (<1E-5)[‡]			NR Hits[§]
					Yeast_ID	Yeast_Gene	Yeast_Description	
FGRRES_04548	FGSG_04548	FGSG_04548		1.98				uncharacterized protein ffuj_10063
FGRRES_05940	FGSG_05940	FGSG_05940		1.98				tob3 (member of aaa-atpase family)
FGRRES_13947	FGSG_13947	FGSG_13947		1.99				cell wall glycoprotein
FGRRES_04881_M	FGSG_04881	FGSG_04881		1.99				cysteine-rich tm module stress tolerance
FGRRES_06993	FGSG_06993	FGSG_06993		1.99				secreted protein
FGRRES_02828	FGSG_02828	FGSG_02828		2.01				uncharacterized protein lw93_4081
FGRRES_07160	FGSG_07160	FGSG_07160		2.02				uncharacterized protein ffuj_02682

FGRRES_10613	FGSG_10613	FGSG_10613	2.04				family protein
FGRRES_03846	FGSG_03846	FGSG_03846	2.04				lipase 4
FGRRES_11079_M	FGSG_11079	FGSG_11079	2.04	YLR004C	THI73	Putative plasma membrane permease; proposed to be involved in carboxylic acid uptake and repressed by thiamine; substrate of Dbf2p/Mob1p kinase; transcription is altered if mitochondrial dysfunction occurs	allantoate permease
FGRRES_13569	FGSG_13569	FGSG_13569	2.05				uncharacterized protein ffuj_04257
FGRRES_03585	FGSG_03585	FGSG_03585	2.05				uncharacterized protein ffuj_10514
FGRRES_05839	FGSG_05839	FGSG_05839	2.05	YDR536W	STL1	Glycerol proton symporter of the plasma membrane; subject to glucose-induced inactivation, strongly but transiently induced when cells are subjected to osmotic shock	sugar transporter stl1
FGRRES_07478	FGSG_07478	FGSG_07478	2.06				uncharacterized protein ffuj_02443
FGRRES_10300	FGSG_10300	FGSG_10300	2.07				uncharacterized protein ffuj_07148
FGRRES_08061_M	FGSG_08601	FGSG_08601	2.07	YKR097W	PCK1	Phosphoenolpyruvate carboxykinase; key enzyme in	phosphoenolpyruvate carboxykinase

						gluconeogenesis, catalyzes early reaction in carbohydrate biosynthesis, glucose represses transcription and accelerates mRNA degradation, regulated by Mcm1p and Cat8p, located in the cytosol	
FGRRES_07821	FGSG_07821	FGSG_07821	2.08				uncharacterized protein lw93_12094
FGRRES_11988	FGSG_11988	FGSG_11988	2.08				abc multidrug
FGRRES_03946	FGSG_03946	FGSG_03946	2.09				thioredoxin
FGRRES_04760_M	FGSG_04760	FGSG_04760	2.10				hypothetical protein FGSG_04760 [Fusarium graminearum PH-1]
FGRRES_10608	FGSG_10608	FGSG_10608	2.10				short-chain dehydrogenase reductase family protein
FGRRES_16777	FGSG_07097	FGSG_07097	2.11				uncharacterized protein lw93_7447
FGRRES_09170	FGSG_09170	FGSG_09170	2.12				uncharacterized protein ffuj_14296
FGRRES_00858	FGSG_00858	FGSG_00858	2.13				uncharacterized protein y057_9795
FGRRES_05908	FGSG_05908	FGSG_05908	2.13				rbtmx2 protein
FGRRES_13106_16799_M	FGSG_13106,FGSG_13107	FGSG_13106,FGSG_13107	2.15				uncharacterized protein ffuj_03191
FGRRES_09146_M	FGSG_09146	FGSG_09146	2.15				ankyrin repeat and socs box protein 7
FGRRES_09889	FGSG_09889	FGSG_09889	2.16				uncharacterized protein ffuj_07572
FGRRES_01496	FGSG_01496	FGSG_01496	2.16				choline-sulfatase
FGRRES_08687	FGSG_08687	FGSG_08687	2.16				uncharacterized protein ffuj_06200
FGRRES_01330	FGSG_01330	FGSG_01330	2.18	YER182W	FMP10	Putative protein of unknown function; the authentic, non-tagged	thioesterase family protein

				protein is detected in highly purified mito-chondria in high-throughput studies	
FGRRES_05291	FGSG_05291	FGSG_05291	2.19		sur7 protein
FGRRES_07588	FGSG_07588	FGSG_07588	2.19		hypothetical protein FGSG_07588 [Fusarium graminearum PH-1]
FGRRES_16462	FGSG_05209	FGSG_05209	2.19		t-complex protein 11
FGRRES_17058	FGSG_08308	FGSG_08308	2.20		multidrug resistance protein
FGRRES_01681	FGSG_01681	FGSG_01681	2.20		atp-grasp domain protein
FGRRES_06612	FGSG_06612	FGSG_06612	2.20		oxalate decarboxylase
FGRRES_03591	FGSG_03591	FGSG_03591	2.22		chitinase 1
FGRRES_16060	FGSG_11565	FGSG_11565	2.24		alkanesulfonate monooxygenase
FGRRES_16301	FGSG_03266	FGSG_03266	2.25		uncharacterized protein ffuj_10888
FGRRES_00016_M	FGSG_00016	FGSG_00016	2.25		unnamed protein product [Fusarium graminearum]
FGRRES_10609	FGSG_10609	FGSG_10609	2.25		fad linked oxidase-like protein
FGRRES_08210	FGSG_08210	FGSG_08210	2.26		hypothetical protein FGSG_08210 [Fusarium graminearum PH-1]
FGRRES_03244	FGSG_03244	FGSG_03244	2.27		enoyl- hydratase
FGRRES_10230	FGSG_10230	FGSG_10230	2.28		4-coumarate-- ligase
FGRRES_00162	FGSG_00162	FGSG_00162	2.28		family oxidoreductase
FGRRES_12862	FGSG_12862	FGSG_12862	2.28		tubulin folding cofactor c
FGRRES_16442	FGSG_04903	FGSG_04903	2.29		cytochrome-b5 reductase
FGRRES_00531	FGSG_00531	FGSG_00531	2.30		family oxidoreductase
FGRRES_16924	FGSG_07904	FGSG_07904	2.32		alternative sulfate transporter
FGRRES_00470	FGSG_00470	FGSG_00470	2.32		cellobiose dehydrogenase
FGRRES_16076_M	FGSG_12203,FGSG_12204	FGSG_12203,FGSG_12204	2.32		tartrate transporter

FGRRES_07852	FGSG_07852	FGSG_07852	2.33	alcohol dehydrogenase bli-4
FGRRES_07390	FGSG_07390	FGSG_07390	2.33	uncharacterized protein ffuj_02532
FGRRES_15687	FGSG_00110	FGSG_00110	2.34	ankyrin repeat protein
FGRRES_08597	FGSG_08597	FGSG_08597	2.35	atp-dependent rna helicase
FGRRES_09650_M	FGSG_09650	FGSG_09650	2.35	uncharacterized protein ffuj_04525
FGRRES_10971	FGSG_10971	FGSG_10971	2.36	protein ybiu
FGRRES_09691	FGSG_09691	FGSG_09691	2.36	c2 domain protein
FGRRES_03144	FGSG_03144	FGSG_03144	2.37	hexose transporter protein
FGRRES_09595	FGSG_09595	FGSG_09595	2.39	aflatoxin efflux pump aflt
FGRRES_11088	FGSG_11088	FGSG_11088	2.41	na(+) h(+) antiporter
FGRRES_10562	FGSG_10562	FGSG_10562	2.41	hypothetical protein FGSG_10562 [Fusarium graminearum PH-1]
FGRRES_16374	FGSG_12560,FGSG_12561	FGSG_12560,FGSG_12561	2.42	ryanodine receptor ryr
FGRRES_17085	FGSG_08205	FGSG_08205	2.42	aminotransferase class-iii
FGRRES_10150	FGSG_10150	FGSG_10150	2.43	ph-response regulator
FGRRES_08292	FGSG_08292	FGSG_08292	2.43	hypothetical protein FGSG_08292 [Fusarium graminearum PH-1]
FGRRES_10483	FGSG_10483	FGSG_10483	2.43	quinate permease
FGRRES_02036	FGSG_02036	FGSG_02036	2.44	uncharacterized protein lw94_318
FGRRES_11162	FGSG_11162	FGSG_11162	2.46	ab hydrolase superfamily protein yisy
FGRRES_17670_M	FGSG_05470	FGSG_05470	2.46	v-type h+-transporting atpase 16kda proteolipid subunit
FGRRES_03619_M	FGSG_03619	FGSG_03619	2.48	unnamed protein product [Fusarium graminearum]
FGRRES_02087	FGSG_02087	FGSG_02087	2.48	uncharacterized protein lw93_5739
FGRRES_05450	FGSG_05450	FGSG_05450	2.48	mercuric reductase
FGRRES_07488	FGSG_07488	FGSG_07488	2.49	uncharacterized protein ffuj_02429
FGRRES_10311	FGSG_10311	FGSG_10311	2.49	uncharacterized protein y057_12149

FGRRES_03563	FGSG_03563	FGSG_03563	2.51	lsb3-possible role in the regulation of actin cytoskeletal organization
FGRRES_11046	FGSG_11046	FGSG_11046	2.51	uncharacterized protein lw93_6101
FGRRES_17449	FGSG_11224	FGSG_11224	2.52	disintegrin and metalloproteinase domain-containing protein b
FGRRES_00738	FGSG_00738	FGSG_00738	2.54	uncharacterized protein ffuj_00499
FGRRES_11985	FGSG_11985	FGSG_11985	2.54	galactose-proton symport
FGRRES_04746	FGSG_04746	FGSG_04746	2.55	uncharacterized protein lw94_11412
FGRRES_13832	FGSG_13832	FGSG_13832	2.56	p-loop containing nucleoside triphosphate partial
FGRRES_05458	FGSG_05458	FGSG_05458	2.56	uncharacterized protein ffuj_08588
FGRRES_11097	FGSG_11097	FGSG_11097	2.56	fungistatic metabolite
FGRRES_16906	FGSG_07820	FGSG_07820	2.58	uncharacterized protein lw93_12095
FGRRES_06548	FGSG_06548	FGSG_06548	2.60	ndp-n-acetyl-d-galactosaminuronic acid dehydrogenase
FGRRES_09522	FGSG_09522	FGSG_09522	2.61	tyrosinase precursor
FGRRES_07988	FGSG_07988	FGSG_07988	2.62	cell wall protein
FGRRES_11864	FGSG_11864	FGSG_11864	2.62	mrna 3 -end-processing protein yth1
FGRRES_08079	FGSG_08079	FGSG_08079	2.63	benzoate 4-monooxygenase
FGRRES_07822	FGSG_07822	FGSG_07822	2.63	serine proteinase
FGRRES_00184	FGSG_00184	FGSG_00184	2.63	xylanase 1
FGRRES_12379_M	FGSG_12379	FGSG_12379	2.64	amino acid transport protein gap1
FGRRES_16907	FGSG_07825	FGSG_07825	2.65	uncharacterized protein lw93_3378
FGRRES_05375	FGSG_05375	FGSG_05375	2.66	betaine-aldehyde dehydrogenase
FGRRES_06549	FGSG_06549	FGSG_06549	2.67	chitin binding protein
FGRRES_10997	FGSG_10997	FGSG_10997	2.68	uncharacterized protein lw93_6944
FGRRES_09267	FGSG_09267	FGSG_09267	2.68	mitochondrial chaperone bcs1
FGRRES_07628	FGSG_07628	FGSG_07628	2.69	3-hydroxybenzoate 6-hydroxylase

FGRRES_03319	FGSG_03319	FGSG_03319	2.71				tob3 (member of aaa-atpase family)
FGRRES_07662_M	FGSG_07662	FGSG_07662	2.72				umta protein
FGRRES_11081	FGSG_11081	FGSG_11081	2.73				zeaxanthin chloroplastic
FGRRES_12331	FGSG_12331	FGSG_12331	2.74				zinc-binding oxidoreductase
FGRRES_05663	FGSG_05663	FGSG_05663	2.77				polysaccharide deacetylase family protein
FGRRES_10386	FGSG_10386	FGSG_10386	2.77				uncharacterized protein ffuj_07062
FGRRES_13464	FGSG_13464	FGSG_13464	2.77				hypothetical protein FGSG_13464 [Fusarium graminearum PH-1]
FGRRES_07096	FGSG_07096	FGSG_07096	2.78				glycerol 3-phosphatase 1
FGRRES_03394	FGSG_03394	FGSG_03394	2.79				secreted protein
FGRRES_01000	FGSG_01000	FGSG_01000	2.80	YHR007C	ERG11	Lanosterol 14-alpha-demethylase; catalyzes C-14 demethylation of lanosterol to form 4,4"-dimethyl cholesta-8,14,24-triene-3-beta-ol in ergosterol biosynthesis pathway; transcriptionally down-regulated when ergosterol is in excess; member of cytochrome P450 family; associated and coordinately regulated with the P450 reductase Ncp1p; human	eburicol 14-alpha-demethylase

				CYP51A1 functionally complements the lethality of the erg11 null mutation	
FGRRES_17390	FGSG_10566	FGSG_10566	2.80		insecticidal toxin complex protein
FGRRES_09493	FGSG_09493	FGSG_09493	2.83		uncharacterized protein ffuj_04359
FGRRES_03659	FGSG_03659	FGSG_03659	2.83		nitrosoguanidine resistance protein sng1
FGRRES_08207_M	FGSG_08207	FGSG_08207	2.84		prostacyclin synthase
FGRRES_04215	FGSG_04215	FGSG_04215	2.84		monocarboxylate transporter 2
FGRRES_17324_M	FGSG_10169	FGSG_10169	2.85		bsc1 transcript encoded by this orf shows a high level of stop codon bypass
FGRRES_16556_M	FGSG_12827	FGSG_12827	2.85		mannitol-1-phosphate 5-dehydrogenase
FGRRES_12651	FGSG_12651	FGSG_12651	2.87		uncharacterized protein ffuj_03895
FGRRES_15753	FGSG_11787	FGSG_11787	2.87		hxb protein
FGRRES_07699	FGSG_07699	FGSG_07699	2.90		hypothetical protein FGSG_07699 [Fusarium graminearum PH-1]
FGRRES_15978	FGSG_02391	FGSG_02391	2.90		uncharacterized protein y057_6261
FGRRES_13505	FGSG_13505	FGSG_13505	2.91		hypothetical protein FGSG_13505 [Fusarium graminearum PH-1]
FGRRES_03554	FGSG_03554	FGSG_03554	2.91		cvnh domain-containing protein
FGRRES_08164	FGSG_08164	FGSG_08164	2.91		transmembrane transporter liz1p
FGRRES_03565	FGSG_03565	FGSG_03565	2.92		nitrogen starvation-induced glutamine rich protein
FGRRES_00023	FGSG_00023	FGSG_00023	2.92		spherulin-1a
FGRRES_17389	FGSG_10564	FGSG_10564	2.93		toxin subunit protein

FGRRES_17232	FGSG_09540	FGSG_09540	2.94				uncharacterized protein lw93_14077
FGRRES_16511	FGSG_05622	FGSG_05622	2.95				trehalase
FGRRES_15926	FGSG_02068	FGSG_02068	2.96				dal81-transcriptional activator
FGRRES_11545	FGSG_11545	FGSG_11545	2.96				uncharacterized protein ffuj_14028
FGRRES_01149	FGSG_01149	FGSG_01149	2.96				ring finger protein b
FGRRES_16063	FGSG_11571	FGSG_11571	3.00	YLL055W	YCT1	High-affinity cysteine-specific transporter; has similarity to the Dal5p family of transporters; green fluorescent protein (GFP)-fusion protein localizes to the endoplasmic reticulum; YCT1 is not an essential gene	cysteine transporter
FGRRES_02925	FGSG_02925	FGSG_02925	3.00				uncharacterized protein ffuj_05402
FGRRES_12434	FGSG_12434	FGSG_12434	3.00				uncharacterized protein y057_15048
FGRRES_16884	FGSG_07719	FGSG_07719	3.00				uncharacterized protein ffuj_14624
FGRRES_01785	FGSG_01785	FGSG_01785	3.01				c4-dicarboxylate transport protein mae1
FGRRES_07861	FGSG_07861	FGSG_07861	3.01				protein cap22
FGRRES_15676	FGSG_11989	FGSG_11989	3.02				aureobasidin a1 biosynthesis complex
FGRRES_04430	FGSG_04430	FGSG_04430	3.02				pectate lyase 1
FGRRES_13422	FGSG_13422	FGSG_13422	3.03				hypothetical protein FGSG_13422 [Fusarium graminearum PH-1]
FGRRES_09072	FGSG_09072	FGSG_09072	3.04				uncharacterized protein lw93_8002
FGRRES_01764	FGSG_01764	FGSG_01764	3.06				hydrophobin
FGRRES_10558	FGSG_10558	FGSG_10558	3.06				tol protein

FGRRES_17361	FGSG_10436	FGSG_10436	3.06	dha14-like major facilitator efflux transporter (mfs transporter)
FGRRES_16183	FGSG_03910	FGSG_03910	3.06	carboxypeptidase y inhibitor
FGRRES_02867	FGSG_02867	FGSG_02867	3.07	kelch domain-containing protein
FGRRES_13795	FGSG_13795	FGSG_13795	3.07	integral membrane protein
FGRRES_10498	FGSG_10498	FGSG_10498	3.07	uncharacterized protein y057_14945
FGRRES_11566_M	FGSG_11566	FGSG_11566	3.08	zinc-binding dehydrogenase
FGRRES_12214	FGSG_12214	FGSG_12214	3.08	uncharacterized protein ffuj_03637
FGRRES_11986	FGSG_11986	FGSG_11986	3.08	fructosyl amino acid
FGRRES_11174_M	FGSG_11174	FGSG_11174	3.09	calcium-independent phospholipase a2-gamma
FGRRES_03725	FGSG_03725	FGSG_03725	3.10	mfs-multidrug-resistance transporter
FGRRES_10565	FGSG_10565	FGSG_10565	3.10	hypothetical protein FGSG_10565 [Fusarium graminearum PH-1]
FGRRES_08201	FGSG_08201	FGSG_08201	3.10	uncharacterized protein y057_3645
FGRRES_10551	FGSG_10551	FGSG_10551	3.12	killer toxin kp4 family protein
FGRRES_06622	FGSG_06622	FGSG_06622	3.12	cycloeucalenol cycloisomerase
FGRRES_10675	FGSG_10675	FGSG_10675	3.14	six-bladed beta-propeller -like protein
FGRRES_01783	FGSG_01783	FGSG_01783	3.14	geranylgeranyl pyrophosphate syn-thase
FGRRES_11475	FGSG_11475	FGSG_11475	3.15	major facilitator superfamily trans-porter
FGRRES_13964	FGSG_13964	FGSG_13964	3.17	c2h2 finger domain containing protein
FGRRES_03801	FGSG_03801	FGSG_03801	3.18	tyrosinase central domain protein
FGRRES_03373	FGSG_03373	FGSG_03373	3.18	alcohol oxidase
FGRRES_02423	FGSG_02423	FGSG_02423	3.19	duf1275 domain protein
FGRRES_02018	FGSG_02018	FGSG_02018	3.20	duf500 domain protein
FGRRES_09142	FGSG_09142	FGSG_09142	3.21	kelch domain-containing protein 8a

FGRRES_12096_7_M	FGSG_12096,FGSG_12097	FGSG_12096,FGSG_12097	3.21				uncharacterized protein lw94_14682
FGRRES_02342_M	FGSG_02342	FGSG_02342	3.21				carbohydrate esterase family 5 protein
FGRRES_11987	FGSG_11987	FGSG_11987	3.22				fad dependent oxidoreductase
FGRRES_03925	FGSG_03925	FGSG_03925	3.22				uncharacterized protein lw94_14686
FGRRES_02145	FGSG_02145	FGSG_02145	3.22	YGR055W	MUP1	High affinity methionine permease; integral membrane protein with 13 putative membrane-spanning regions; involved in both methionine and cysteine uptake; subject to transport-activity dependent, methionine-induced endocytosis, vacuolar delivery and degradation; starvation-induced endocytosis is transport-activity independent	amino acid permease
FGRRES_03432	FGSG_03432	FGSG_03432	3.22				endothiapepsin precursor
FGRRES_03674_M	FGSG_03674	FGSG_03674	3.23				filamentous hemagglutinin adhesin
FGRRES_09103	FGSG_09103	FGSG_09103	3.23				2og-fe oxygenase superfamily protein
FGRRES_04429	FGSG_04429	FGSG_04429	3.23				uncharacterized protein lw93_9003
FGRRES_12335	FGSG_12335	FGSG_12335	3.24				dha14-like major facilitator efflux transporter (mfs transporter)
FGRRES_17148_M	FGSG_09029	FGSG_09029	3.25				uncharacterized protein lw93_5233

FGRRES_10636	FGSG_10636	FGSG_10636	3.26	translationally-controlled tumor protein
FGRRES_03873_M	FGSG_03783	FGSG_03783	3.26	uncharacterized protein lw93_7023
FGRRES_07810	FGSG_07810	FGSG_07810	3.27	n amino acid transport system protein
FGRRES_04667	FGSG_04667	FGSG_04667	3.27	alpha-ketoglutarate-dependent sulfonate dioxygenase
FGRRES_03714	FGSG_03714	FGSG_03714	3.28	dfg5 protein
FGRRES_09289	FGSG_09289	FGSG_09289	3.30	ice nucleation protein
FGRRES_16872	FGSG_07664	FGSG_07664	3.35	unnamed protein product [Fusarium graminearum]
FGRRES_16093	FGSG_04566	FGSG_04566	3.36	uncharacterized protein y057_13136
FGRRES_17149	FGSG_09033	FGSG_09033	3.36	uncharacterized protein y057_3621
FGRRES_11568	FGSG_11568	FGSG_11568	3.37	4-hydroxyacetophenone monooxygenase
FGRRES_02668	FGSG_02668	FGSG_02668	3.38	psi-producing oxygenase c
FGRRES_10326	FGSG_10326	FGSG_10326	3.38	uncharacterized protein ffuj_07122
FGRRES_13423	FGSG_13423	FGSG_13423	3.40	hypothetical protein FGSG_13423 [Fusarium graminearum PH-1]
FGRRES_01342	FGSG_01342	FGSG_01342	3.40	alternative mitochondrial
FGRRES_09477	FGSG_09477	FGSG_09477	3.41	related to fad-binding
FGRRES_08021_M	FGSG_08021	FGSG_08021	3.42	cell surface protein
FGRRES_13952	FGSG_13952	FGSG_13952	3.43	uncharacterized protein ffuj_08920
FGRRES_03120	FGSG_03120	FGSG_03120	3.43	diacylglycerol pyrophosphate phosphatase dpp1
FGRRES_05846	FGSG_05846	FGSG_05846	3.46	uncharacterized protein lw94_5257
FGRRES_11080	FGSG_11080	FGSG_11080	3.47	integral membrane protein
FGRRES_12497	FGSG_12497	FGSG_12497	3.50	heterokaryon incompatibility
FGRRES_17057	FGSG_13393,FGSG_13394	FGSG_13393,FGSG_13394	3.52	long-chain-fatty-acid- ligase

FGRRES_16175	FGSG_03973	FGSG_03973	3.53				serum paraoxonase arylesterase
FGRRES_07567	FGSG_07567	FGSG_07567	3.54				multidrug resistance protein
FGRRES_02154_M	FGSG_02154	FGSG_02154	3.54				unnamed protein product [Fusarium graminearum]
FGRRES_01513	FGSG_01513	FGSG_01513	3.54				uncharacterized protein lw93_11502
FGRRES_16581	FGSG_05967	FGSG_05967	3.56				uncharacterized protein ffuj_13160
FGRRES_11155	FGSG_11155	FGSG_11155	3.57				hypothetical protein FGSG_11155 [Fusarium graminearum PH-1]
FGRRES_07027	FGSG_07027	FGSG_07027	3.57				uncharacterized protein ffuj_02827
FGRRES_03245_M	FGSG_03245	FGSG_03245	3.58				non-ribosomal peptide synthetase
FGRRES_02748	FGSG_02748	FGSG_02748	3.58				endothelin-converting enzyme 1
FGRRES_00209	FGSG_00209	FGSG_00209	3.58				uncharacterized protein ffuj_10847
FGRRES_03588	FGSG_03588	FGSG_03588	3.61				integral membrane protein pth11
FGRRES_12424	FGSG_12424	FGSG_12424	3.61				uncharacterized protein ffuj_10439
FGRRES_12445	FGSG_12445	FGSG_12445	3.64				inactive purple acid phosphatase 16
FGRRES_03586	FGSG_03586	FGSG_03586	3.64				uncharacterized protein y057_6275
FGRRES_03489	FGSG_03489	FGSG_03489	3.64				uncharacterized protein lw93_10986
FGRRES_07532_M	FGSG_07532	FGSG_07532	3.65				integral membrane protein
FGRRES_10081	FGSG_10081	FGSG_10081	3.66				hard surface induced protein 3
FGRRES_03233	FGSG_03233	FGSG_03233	3.67				sugar transporter
FGRRES_07389	FGSG_07389	FGSG_07389	3.70	YNR074C	AIF1	Mitochondrial cell death effector; translocates to the nucleus in response to apoptotic stimuli; triggers apoptosis in cells lacking HXK2; homolog of mammalian	uncharacterized protein ffuj_02531

				Apoptosis-Inducing Factor, putative reductase	
FGRRES_12123	FGSG_12123	FGSG_12123	3.70		siderophore biosynthesis
FGRRES_13432	FGSG_13432	FGSG_13432	3.71		uncharacterized protein ffuj_11541
FGRRES_12251	FGSG_12251	FGSG_12251	3.72		arylsulfatase
FGRRES_07569	FGSG_07569	FGSG_07569	3.74		rf2 protein
FGRRES_05838	FGSG_05838	FGSG_05838	3.74		uncharacterized protein y057_13444
FGRRES_03587	FGSG_03587	FGSG_03587	3.75		uncharacterized protein y057_6261
FGRRES_01661	FGSG_01661	FGSG_01661	3.77		taurine dioxygenase
FGRRES_16869	FGSG_07647	FGSG_07647	3.80		uncharacterized protein lw93_6396
FGRRES_02933	FGSG_02933	FGSG_02933	3.81		uncharacterized protein y057_10495
FGRRES_03620	FGSG_03620	FGSG_03620	3.82		hypothetical protein FGSG_03620 [Fusarium graminearum PH-1]
FGRRES_02269	FGSG_02269	FGSG_02269	3.83		uncharacterized protein ffuj_03548
FGRRES_03828	FGSG_03828	FGSG_03828	3.84		mannose-binding lectin
FGRRES_00725	FGSG_00725	FGSG_00725	3.84		c6 transcription factor
FGRRES_06517	FGSG_06517	FGSG_06517	3.84		dihydroxy-acid dehydratase
FGRRES_06149	FGSG_06149	FGSG_06149	3.87		diatom spindle kinesin 1
FGRRES_03748	FGSG_03748	FGSG_03748	3.89		uncharacterized protein ffuj_10848
FGRRES_04600	FGSG_04600	FGSG_04600	3.89		hypothetical protein FGSG_04600 [Fusarium graminearum PH-1]
FGRRES_11341	FGSG_11341	FGSG_11341	3.95		udp-glycosyltransferase 84b2
FGRRES_12815	FGSG_12815	FGSG_12815	3.97		hypothetical protein FGSG_12815 [Fusarium graminearum PH-1]
FGRRES_16332	FGSG_03091	FGSG_03091	3.97		uncharacterized protein ffuj_11840
FGRRES_11630	FGSG_11630	FGSG_11630	3.97		uncharacterized protein ffuj_05664
FGRRES_03812	FGSG_03812	FGSG_03812	4.01		epoxide hydrolase

FGRRES_02137	FGSG_02137	FGSG_02137	4.01	cytochrome-b5 reductase
FGRRES_11436	FGSG_11436	FGSG_11436	4.02	uncharacterized protein lw93_14918
FGRRES_03695	FGSG_03695	FGSG_03695	4.02	endoglucanase iv precursor
FGRRES_11146	FGSG_11146	FGSG_11146	4.02	heme peroxidase
FGRRES_03001	FGSG_03001	FGSG_03001	4.02	uncharacterized protein ffuj_11899
FGRRES_11461	FGSG_11461	FGSG_11461	4.03	uncharacterized protein lw93_3994
FGRRES_02667	FGSG_02667	FGSG_02667	4.05	uncharacterized protein y057_4108
FGRRES_07895	FGSG_07895	FGSG_07895	4.05	uncharacterized protein ffuj_14529
FGRRES_02217	FGSG_02217	FGSG_02217	4.08	catalase
FGRRES_12956	FGSG_12956	FGSG_12956	4.14	uncharacterized protein lw93_1732
FGRRES_03361_M	FGSG_03361	FGSG_03361	4.16	transcriptional regulatory protein
FGRRES_03046	FGSG_03046	FGSG_03046	4.17	uncharacterized protein y057_2145
FGRRES_01778	FGSG_01778	FGSG_01778	4.18	hypothetical protein FGSG_01778 [Fusarium graminearum PH-1]
FGRRES_05719	FGSG_05719	FGSG_05719	4.18	uncharacterized protein lw93_11922
FGRRES_09086_M	FGSG_09086	FGSG_09086	4.19	unnamed protein product [Fusarium graminearum]
FGRRES_17059	FGSG_08302	FGSG_08302	4.23	rna polymerase rpb1 c-terminal repeat domain protein
FGRRES_09311	FGSG_09311	FGSG_09311	4.23	phenylalanine ammonia-lyase
FGRRES_03042	FGSG_03042	FGSG_03042	4.24	2`-hydroxyisoflavone reductase
FGRRES_16074	FGSG_04684	FGSG_04684	4.28	uncharacterized protein lw93_6843
FGRRES_01126	FGSG_01126	FGSG_01126	4.29	peptidoglycan binding domain-containing protein
FGRRES_11204	FGSG_11204	FGSG_11204	4.30	secreted protein
FGRRES_02398	FGSG_02398	FGSG_02398	4.31	basic-leucine zipper transcription factor

FGRRES_02125	FGSG_02125	FGSG_02125	4.32		tgf beta induced protein ig-h3 precursor
FGRRES_17340	FGSG_13739	FGSG_13739	4.32		benzoate 4-monooxygenase cytochrome
FGRRES_03680	FGSG_03680	FGSG_03680	4.32		aquaporin rerated other eukaryote
FGRRES_03101	FGSG_03101	FGSG_03101	4.34		uncharacterized protein ffuj_11665
FGRRES_09521	FGSG_09521	FGSG_09521	4.38		dihydrodipicolinate synthetase
FGRRES_07318	FGSG_07318	FGSG_07318	4.39		adh5-alcohol dehydrogenase v
FGRRES_03832	FGSG_03832	FGSG_03832	4.39		related to ankyrin
FGRRES_03040	FGSG_03040	FGSG_03040	4.39		uncharacterized protein lw93_3388
FGRRES_04442	FGSG_04442	FGSG_04442	4.39		uncharacterized protein ffuj_14701
FGRRES_05799	FGSG_05799	FGSG_05799	4.40		uncharacterized protein lw93_8185
FGRRES_08076	FGSG_08076	FGSG_08076	4.41		uncharacterized protein cpur_03265
FGRRES_16280	FGSG_03388	FGSG_03388	4.42		uncharacterized protein lw93_7820
FGRRES_16506_M	FGSG_05588	FGSG_05588	4.43		uncharacterized protein ffuj_08721
FGRRES_11192	FGSG_11192	FGSG_11192	4.49		l-amino acid oxidase
FGRRES_17122	FGSG_13461	FGSG_13461	4.50		related to integral membrane protein pth11
FGRRES_16061	FGSG_11567	FGSG_11567	4.60		major facilitator superfamily transporter
FGRRES_11132	FGSG_11132	FGSG_11132	4.63		cyanamide hydratase
FGRRES_01695_M	FGSG_01695	FGSG_01695	4.64		uncharacterized protein ffuj_01909
FGRRES_17404	FGSG_10621	FGSG_10621	4.67		xyloglucan endo-transglycosylase-like protein
FGRRES_03521	FGSG_03521	FGSG_03521	4.67		cupin superfamily protein
FGRRES_12673	FGSG_12673	FGSG_12673	4.68		hypothetical protein FGSG_12673 [Fusarium graminearum PH-1]
FGRRES_12738	FGSG_12738	FGSG_12738	4.68		uncharacterized protein ffuj_14917

FGRRES_05076	FGSG_05076	FGSG_05076	4.69				abc transporter protein (atp-binding-cassette protein)
FGRRES_02486	FGSG_02486	FGSG_02486	4.70				gmp synthase
FGRRES_16115_M	FGSG_04440	FGSG_04440	4.72				multidrug transporter
FGRRES_05606	FGSG_05606	FGSG_05606	4.74				uncharacterized protein ffuj_08741
FGRRES_03030_M	FGSG_03030	FGSG_03030	4.78				unnamed protein product [Fusarium graminearum]
FGRRES_07578	FGSG_07578	FGSG_07578	4.79				3-dehydroquinate synthase
FGRRES_17553	FGSG_11422	FGSG_11422	4.84				phosphatidylserine decarboxylase 2
FGRRES_09133	FGSG_09133	FGSG_09133	4.85				uncharacterized protein ffuj_10134
FGRRES_12523	FGSG_12523	FGSG_12523	4.86				transmembrane protein 53-b
FGRRES_16049	FGSG_11529	FGSG_11529	4.88				integral membrane protein
FGRRES_03274	FGSG_03274	FGSG_03274	4.88				uncharacterized protein lw93_15001
FGRRES_04797	FGSG_04797	FGSG_04797	4.91				uncharacterized protein ffuj_11303
FGRRES_07741_M	FGSG_07741	FGSG_07741	4.94				volvatoxin a2 precursor
FGRRES_07388	FGSG_07388	FGSG_07388	4.97				enoyl- hydratase isomerase
FGRRES_16103	FGSG_12237,FGSG_12239	FGSG_12237,FGSG_12239	4.97				ankyrin repeat protein
FGRRES_11166	FGSG_11166	FGSG_11166	4.97				hypothetical protein FGSG_11166 [Fusarium graminearum PH-1]
FGRRES_09336	FGSG_09336	FGSG_09336	4.98				uncharacterized protein ffuj_12690
FGRRES_03819	FGSG_03819	FGSG_03819	4.98	YOR246C	ENV9	Conserved oxidoreductase involved in lipid droplet morphology; mutant shows defects in CPY processing and vacuolar morphology; required for replication of	related to reductases

						Brome mosaic virus in S. cerevisiae, a model system for studying replication of positive-strand RNA viruses in their natural hosts; homologus to human RDH12 linked to Leber Congenital Amaurosis	
FGRRES_10612	FGSG_10612	FGSG_10612	4.99				salicylate hydroxylase
FGRRES_03275	FGSG_03275	FGSG_03275	4.99				uncharacterized protein ffuj_14309
FGRRES_00003	FGSG_00003	FGSG_00003	5.01				uncharacterized protein lw93_11030
FGRRES_16845	FGSG_07556	FGSG_07556	5.01				sulphydryl oxidase
FGRRES_16226	FGSG_12399,FGSG_12400	FGSG_12399,FGSG_12400	5.03				cercosporin resistance protein
FGRRES_16205	FGSG_03782	FGSG_03782	5.06				6-hydroxy-d-nicotine oxidase
FGRRES_09054	FGSG_09054	FGSG_09054	5.07				flavin-binding monooxygenase-like family protein
FGRRES_03463	FGSG_03463	FGSG_03463	5.07				uncharacterized protein y057_2935
FGRRES_02861	FGSG_02861	FGSG_02861	5.08				uncharacterized protein ffuj_05524
FGRRES_10052	FGSG_10052	FGSG_10052	5.09				integral membrane protein
FGRRES_04745	FGSG_04745	FGSG_04745	5.10				antifungal protein
FGRRES_10623	FGSG_10623	FGSG_10623	5.15				uncharacterized protein ffuj_12690
FGRRES_04029	FGSG_04029	FGSG_04029	5.16				uncharacterized protein ffuj_09454
FGRRES_03167_M	FGSG_03167	FGSG_03167	5.17				uncharacterized protein ffuj_11017
FGRRES_06656	FGSG_06656	FGSG_06656	5.19	YOR049C	RSB1	Sphingoid long-chain base (LCB) efflux transporter; integral	sphingoid long-chain base transporter rsb1

				membrane transporter that localizes to the plasma membrane and may transport long chain bases (LCBs) from the cytoplasmic side toward the extra-cytoplasmic side of the membrane; role in glycerophospholipid translocation; suppres-sor of the sphingoid LCB sensitivity of an LCB-lyase mutation	
FGRRES_09104_M	FGSG_09104	FGSG_09104	5.23		pantothenate transporter liz1
FGRRES_03613	FGSG_03613	FGSG_03613	5.24		uncharacterized protein ffuj_10585
FGRRES_00734	FGSG_00734	FGSG_00734	5.27		uncharacterized protein lw93_8075
FGRRES_07582	FGSG_07582	FGSG_07582	5.28		mfs sp sugar:h+ symporter
FGRRES_07982_M	FGSG_07982	FGSG_07982	5.29		unnamed protein product [Fusarium graminearum]
FGRRES_16013	FGSG_02550,FGSG_02551	FGSG_02550,FGSG_02551	5.31		manganese peroxidase 2
FGRRES_02138	FGSG_02138	FGSG_02138	5.35		cytochrome p450 oxidoreductase
FGRRES_13979	FGSG_13979	FGSG_13979	5.35		gnat family acetyltransferase
FGRRES_06783	FGSG_06783	FGSG_06783	5.36		uncharacterized protein ffuj_14896
FGRRES_17531	FGSG_13938	FGSG_13938	5.38		probable modin
FGRRES_15948	FGSG_02196	FGSG_02196	5.38		uncharacterized protein ffuj_02288
FGRRES_03788	FGSG_03788	FGSG_03788	5.39		glycoside hydrolase family 16 protein
FGRRES_17587	FGSG_11242	FGSG_11242	5.52		aerobactin siderophore

FGRRES_16426	FGSG_12626,FGSG_12627	FGSG_12626,FGSG_12627	5.55	ankyrin repeat domain-containing protein 17
FGRRES_01784	FGSG_01784	FGSG_01784	5.59	cral trio domain protein
FGRRES_03182	FGSG_03182	FGSG_03182	5.66	tubulin-tyrosine ligase
FGRRES_00876	FGSG_00876	FGSG_00876	5.67	protein tos1
FGRRES_15875	FGSG_11990	FGSG_11990	5.68	uncharacterized protein ffuj_01907
FGRRES_04796	FGSG_04796	FGSG_04796	5.73	hypothetical protein FGSG_04796 [Fusarium graminearum PH-1]
FGRRES_10583	FGSG_10583	FGSG_10583	5.73	uncharacterized protein lw93_13170
FGRRES_16114	FGSG_04440	FGSG_04440	5.75	multidrug transporter
FGRRES_11499	FGSG_11499	FGSG_11499	5.78	alpha- -mannosyl-glycoprotein 4-beta-n-acetylglucosaminyltransferase c
FGRRES_11316	FGSG_11316	FGSG_11316	5.84	alcohol dehydrogenase -like domain-containing protein
FGRRES_07207	FGSG_07207	FGSG_07207	5.90	glycosyl hydrolase family 43 protein
FGRRES_00072	FGSG_00072	FGSG_00072	5.92	uncharacterized protein ffuj_03953
FGRRES_10560	FGSG_10560	FGSG_10560	5.94	hypothetical protein FGSG_10560 [Fusarium graminearum PH-1]
FGRRES_10998	FGSG_10998	FGSG_10998	5.97	6-hydroxy-d-nicotine oxidase
FGRRES_00161	FGSG_00161	FGSG_00161	6.02	high-affinity methionine permease
FGRRES_00014_M	FGSG_00014	FGSG_00014	6.03	polysaccharide synthase
FGRRES_08244	FGSG_08244	FGSG_08244	6.08	uncharacterized protein y057_13678
FGRRES_15917_M	FGSG_12047	FGSG_12047	6.13	endo- -beta-xylanase
FGRRES_11396	FGSG_11396	FGSG_11396	6.13	asparagine synthase
FGRRES_04810	FGSG_04810	FGSG_04810	6.13	hypothetical protein FGSG_04810 [Fusarium graminearum PH-1]
FGRRES_04749	FGSG_04749	FGSG_04749	6.16	integral membrane protein pth11
FGRRES_00260	FGSG_00260	FGSG_00260	6.16	uncharacterized protein ffuj_00360

FGRRES_03907	FGSG_03907	FGSG_03907	6.23				uncharacterized protein y057_12670
FGRRES_04188	FGSG_04188	FGSG_04188	6.30				hc-toxin efflux carrier toxa
FGRRES_16358	FGSG_02981	FGSG_02981	6.33				hypothetical protein FG05_02981 [Fusarium graminearum]
FGRRES_04717	FGSG_04717	FGSG_04717	6.34				ent-kaurene oxidase
FGRRES_15872	FGSG_01680	FGSG_01680	6.35				nonribosomal peptide
FGRRES_01862	FGSG_01862	FGSG_01862	6.41				uncharacterized protein ffuj_12176
FGRRES_06541	FGSG_06541	FGSG_06541	6.48				integral membrane protein
FGRRES_15983	FGSG_02399	FGSG_02399	6.55				aur protein kinase
FGRRES_14010	FGSG_14010	FGSG_14010	6.68				hypothetical protein FGSG_14010 [Fusarium graminearum PH-1]
FGRRES_10464	FGSG_10464	FGSG_10464	6.86				polyketide synthase
FGRRES_10540	FGSG_10540	FGSG_10540	6.95				hypothetical protein FGSG_10540 [Fusarium graminearum PH-1]
FGRRES_17532	FGSG_13939	FGSG_13939	6.97				unnamed protein product [Fusarium graminearum]
FGRRES_07774	FGSG_07774	FGSG_07774	6.99				uncharacterized protein ffuj_10880
FGRRES_08114_M	FGSG_08114	FGSG_08114	7.04				5-methylthioadenosine s-adenosylho-mocysteine deaminase
FGRRES_05796	FGSG_05796	FGSG_05796	7.09				ankyrin repeat protein
FGRRES_04795	FGSG_04795	FGSG_04795	7.11				hypothetical protein FGSG_04795 [Fusarium graminearum PH-1]
FGRRES_16173	FGSG_12336	FGSG_12336	7.21				phosphoenolpyruvate phosphomutase
FGRRES_11647	FGSG_11647	FGSG_11647	7.30				hypothetical protein FGSG_11647 [Fusarium graminearum PH-1]
FGRRES_17545	FGSG_11450	FGSG_11450	7.35				tyrosinase
FGRRES_07557	FGSG_07557	FGSG_07557	7.44	YML051W	GAL80	Transcriptional regula-tor involved in the	transcription co-repressor gal80

				repression of GAL genes; involved in the repression of GAL genes in the absence of galactose; inhibits transcriptional activation by Gal4p; inhibition relieved by Gal3p or Gal1p binding	
FGRRES_11546	FGSG_11546	FGSG_11546	7.53		uncharacterized protein y057_1052
FGRRES_03768	FGSG_03768	FGSG_03768	7.56		uncharacterized protein ffuj_11446
FGRRES_07755	FGSG_07755	FGSG_07755	7.57		pathogenicity protein
FGRRES_02216	FGSG_02216	FGSG_02216	7.58		lipoxygenase 1
FGRRES_04850	FGSG_04850	FGSG_04850	7.62		hypothetical protein FGSG_04850 [Fusarium graminearum PH-1]
FGRRES_16864	FGSG_07627	FGSG_07627	7.65		6-hydroxy-d-nicotine oxidase
FGRRES_10559	FGSG_10559	FGSG_10559	7.74		hypothetical protein FGSG_10559 [Fusarium graminearum PH-1]
FGRRES_17550	FGSG_11427	FGSG_11427	8.06		laccase 2
FGRRES_00060	FGSG_00060	FGSG_00060	8.13		killer kp4 smk- core
FGRRES_10618	FGSG_10618	FGSG_10618	8.13		hypothetical protein FGSG_10618 [Fusarium graminearum PH-1]
FGRRES_02935	FGSG_02935	FGSG_02935	8.42		hypothetical protein FGSG_02935 [Fusarium graminearum PH-1]
FGRRES_15678_M	FGSG_00020	FGSG_00020	8.48		unnamed protein product [Fusarium graminearum]
FGRRES_16423	FGSG_04825	FGSG_04825	8.53		integral membrane protein
FGRRES_03981_M	FGSG_03981	FGSG_03981	8.61		aspartate aminotransferase

FGRRES_13865	FGSG_13865	FGSG_13865	8.66	aldehyde dehydrogenase
FGRRES_10474	FGSG_10474	FGSG_10474	8.71	plastidic glucose transporter 4
FGRRES_10098	FGSG_10098	FGSG_10098	8.72	mfs-multidrug-resistance transporter
FGRRES_09345	FGSG_09345	FGSG_09345	8.77	hypothetical protein FGSG_09345 [Fusarium graminearum PH-1]
FGRRES_03811	FGSG_03811	FGSG_03811	8.83	hypothetical protein FGSG_03811 [Fusarium graminearum PH-1]
FGRRES_12240	FGSG_12240	FGSG_12240	8.92	small s protein
FGRRES_00224	FGSG_00224	FGSG_00224	9.05	uncharacterized protein lw93_14870
FGRRES_11222	FGSG_11222	FGSG_11222	9.19	hypothetical protein FGSG_11222 [Fusarium graminearum PH-1]
FGRRES_04743	FGSG_04743	FGSG_04743	9.20	alpha-l-rhamnosidase c
FGRRES_02294	FGSG_02294	FGSG_02294	9.35	uncharacterized protein ffuj_14613
FGRRES_00062	FGSG_00062	FGSG_00062	9.44	killer kp4 smk- core
FGRRES_16154	FGSG_04078	FGSG_04078	9.48	uncharacterized protein y057_217
FGRRES_03096	FGSG_03096	FGSG_03096	9.50	hypothetical protein FGSG_03096 [Fusarium graminearum PH-1]
FGRRES_15709	FGSG_00221	FGSG_00221	9.53	unnamed protein product [Fusarium graminearum]
FGRRES_12124_15979_M	FGSG_12124,FGSG_12125	FGSG_12124,FGSG_12125	9.58	k+ channel protein
FGRRES_11399	FGSG_11399	FGSG_11399	9.62	fad binding domain-containing protein
FGRRES_13827	FGSG_13827	FGSG_13827	9.86	conserved uncharacterized protein
FGRRES_10458	FGSG_10458	FGSG_10458	9.94	c6 transcription factor
FGRRES_16989_M	FGSG_06989	FGSG_06989	10.00	protein serac1
FGRRES_04518	FGSG_04518	FGSG_04518	10.21	uncharacterized protein y057_10431
FGRRES_10561	FGSG_10561	FGSG_10561	10.26	glycoside hydrolase family 18 protein
FGRRES_10563_M	FGSG_10563	FGSG_10563	10.41	uncharacterized protein y057_732
FGRRES_11368	FGSG_11368	FGSG_11368	10.64	mannose-binding lectin

FGRRES_02645	FGSG_02645	FGSG_02645	10.78			micromolar calcium activated neutral protease 1
FGRRES_10191	FGSG_10191	FGSG_10191	10.85	YML079W	Non-essential protein of unknown function; has structural resemblance to plant storage and ligand binding proteins (canavalin, glycinin, auxin binding protein) and to some enzymes (epimerase, germin); localizes to the nucleus and cytoplasm	duf985 domain protein
FGRRES_13109	FGSG_13109	FGSG_13109	10.88			uncharacterized protein ffuj_03177
FGRRES_06462	FGSG_06462	FGSG_06462	10.93			coenzyme a synthetase
FGRRES_10395	FGSG_10395	FGSG_10395	11.38			laccase precursor
FGRRES_17125	FGSG_07989	FGSG_07989	12.19			unnamed protein product [Fusarium graminearum]
FGRRES_03775	FGSG_03775	FGSG_03775	12.20			zeaxanthin chloroplastic
FGRRES_04624	FGSG_04624	FGSG_04624	12.21			hypothetical protein FGSG_04624 [Fusarium graminearum PH-1]
FGRRES_04740	FGSG_04740	FGSG_04740	12.28			hypothetical protein FGSG_04740 [Fusarium graminearum PH-1]
FGRRES_11032	FGSG_11032	FGSG_11032	12.35			galactose oxidase
FGRRES_17438	FGSG_13809,FGSG_13810	FGSG_13809,FGSG_13810	12.68			membrane attack complex component perforin
FGRRES_04450	FGSG_04450	FGSG_04450	13.27			uncharacterized protein ffuj_09932

FGRRES_16373_M	FGSG_02894	FGSG_02894	13.88	sarcosine oxidase
FGRRES_09570	FGSG_09570	FGSG_09570	13.89	uncharacterized protein lw93_14043
FGRRES_05589	FGSG_05589	FGSG_05589	13.97	atp-binding cassette protein
FGRRES_17151	FGSG_09046	FGSG_09046	14.04	uncharacterized protein lw94_9396
FGRRES_09372	FGSG_09372	FGSG_09372	14.80	nitrogen metabolic regulation protein nmr
FGRRES_13433	FGSG_13433	FGSG_13433	15.18	uncharacterized protein lw94_13071
FGRRES_09187	FGSG_09187	FGSG_09187	15.27	fusarubin cluster-dehydrogenase
FGRRES_04019	FGSG_04019	FGSG_04019	15.54	uncharacterized protein y057_9957
FGRRES_17101_M	FGSG_08108	FGSG_08108	16.17	ankyrin repeat protein
FGRRES_16849_M	FGSG_08108	FGSG_08108	16.18	seed maturation protein pm25
FGRRES_17439	FGSG_10700	FGSG_10700	16.26	membrane attack complex component perforin
FGRRES_10604	FGSG_10604	FGSG_10604	17.89	laccase precursor
FGRRES_10629	FGSG_10629	FGSG_10629	18.63	tri13-cytochrome p450
FGRRES_03969	FGSG_03969	FGSG_03969	19.71	uncharacterized protein ffuj_09374
FGRRES_00114	FGSG_00114	FGSG_00114	19.88	uncharacterized protein ffuj_03936

Table S4. Analysis DON production in the *Fgsfl1* mutant.

Strain	DON/ppm[a,b]
PH-1	1130.1±303.0[A]
SF-1	1063.9±264.0[A]
CS-1	955.7±122.9[A]

[a] Standard deviation (mean ± standard deviation) were calculated from at least three independent measurements. [b]Flowering wheat heads were inoculated and infected wheat kernels were harvested for DON assays 14 dpi.

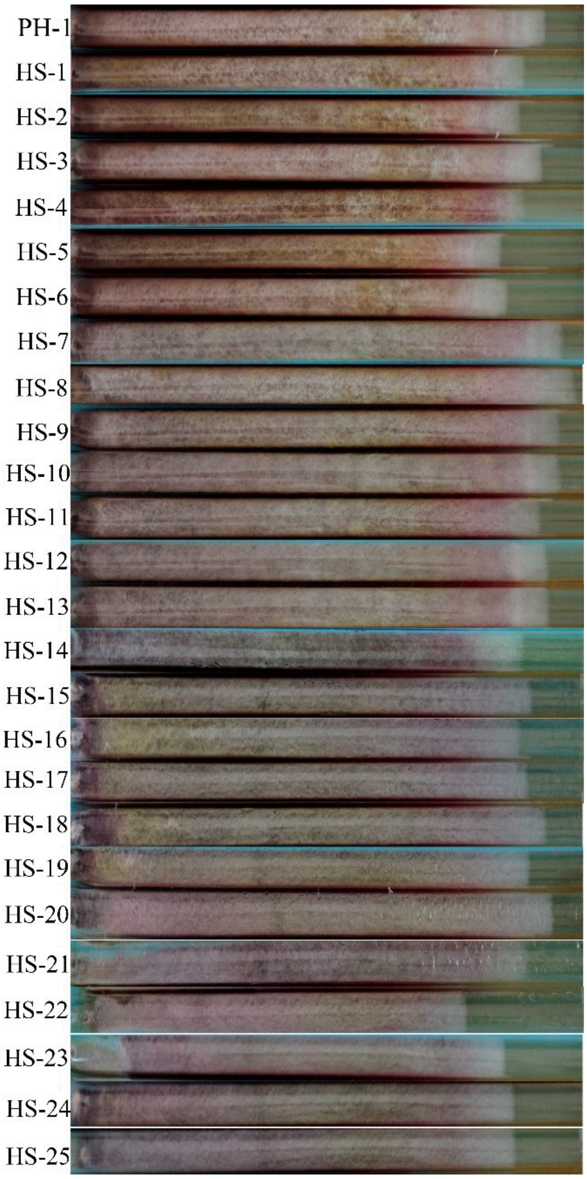

Figure S1. Growth of spontaneous suppressor strains in race tube after cultivation for 15 days.

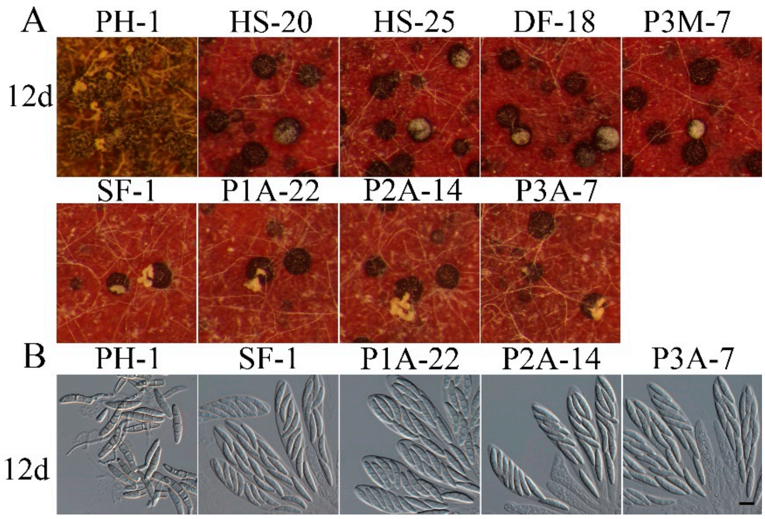

Figure S2. Analysis of ascospores discharge from HS-20, HS-25, DF-18, SF-1, P1A-22, P2A-14, and P3A-7 on carrot plate for 12 days. **A.** Carrot agar cultures of the wild-type strain PH-1, HS-20, HS-25, SF-1, DF-18, P1A-22, P2A-14, and P3A-7 were examined 7 days post-fertilization (dpf) and assay of perithecia of the wild-type strain PH-1, HS-20, HS-25, SF-1, DF-18, P1A-22, P2A-14, and P3A-7. **B.** Ascospores of the wild-type strain PH-1, SF-1, P1A-22, P2A-14, and P3A-7 were examined by differential interference contrast (DIC). Bar= 10 μm.

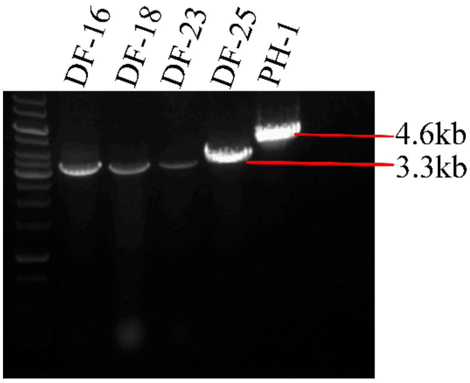

Figure S3. Putative *sfl1 cpkA cpk2* mutants were screened by PCR analysis. The target DNA stripes were amplified with FgSFL1-7F- FgSFL1-8R. Putative *Fgsfl1* mutants were identified (DF-16, DF-18, DF-23, DF-25).

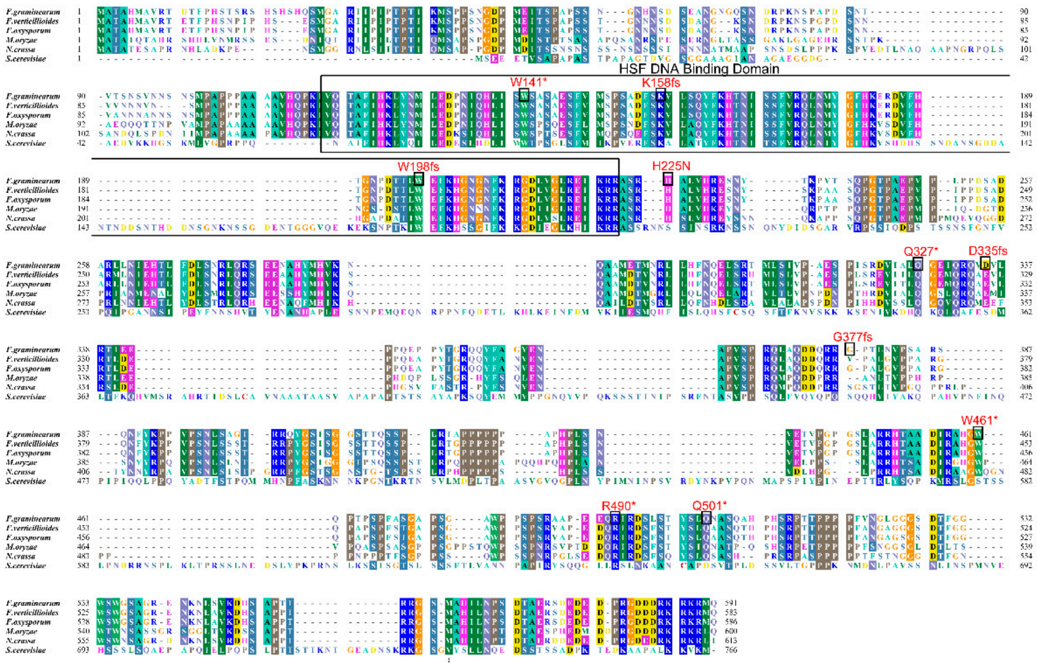

Figure S4. Sequence alignment of *SFL1*. Sequence of the selected fungal *SFL1* orthologues were aligned by Clustal X and viewed by BioEdit program. Identical amino acids are highlighted in the same color, conserved HSF binding domains is boxed in black. Amino acid changes in *SFL1* identified in 25 suppressor strains are labeled above the sequence alignments in red. The compared sequences were from *F. graminearum*, *S. cerevisiae*, *M. oryzae*, *N. crassa*, *F. pseudograminearum*, *F. oxysporum* and *F. verticillioides*.

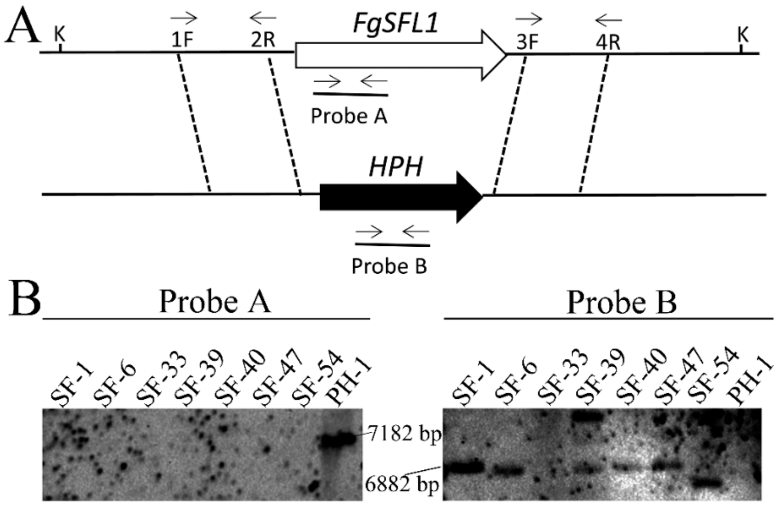

Figure S5. Putative *sfl1* mutants were screened by southern blot analysis. A. Schematic diagram of southern blot. B. Southern blot of *Fgsfl1* mutant.

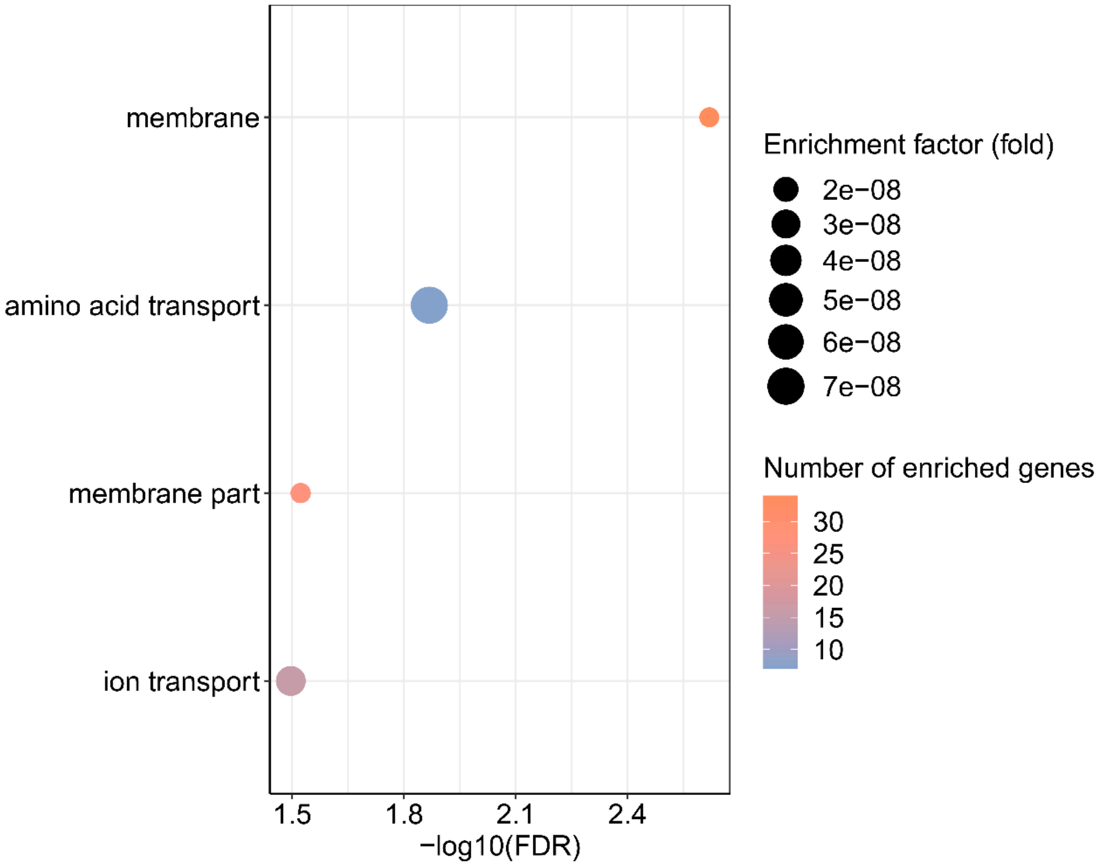

Figure S6. GO analysis of up-regulated genes in the 12-hours hyphae of the wild-type strain PH-1 and the *Fgsfl1* mutant.

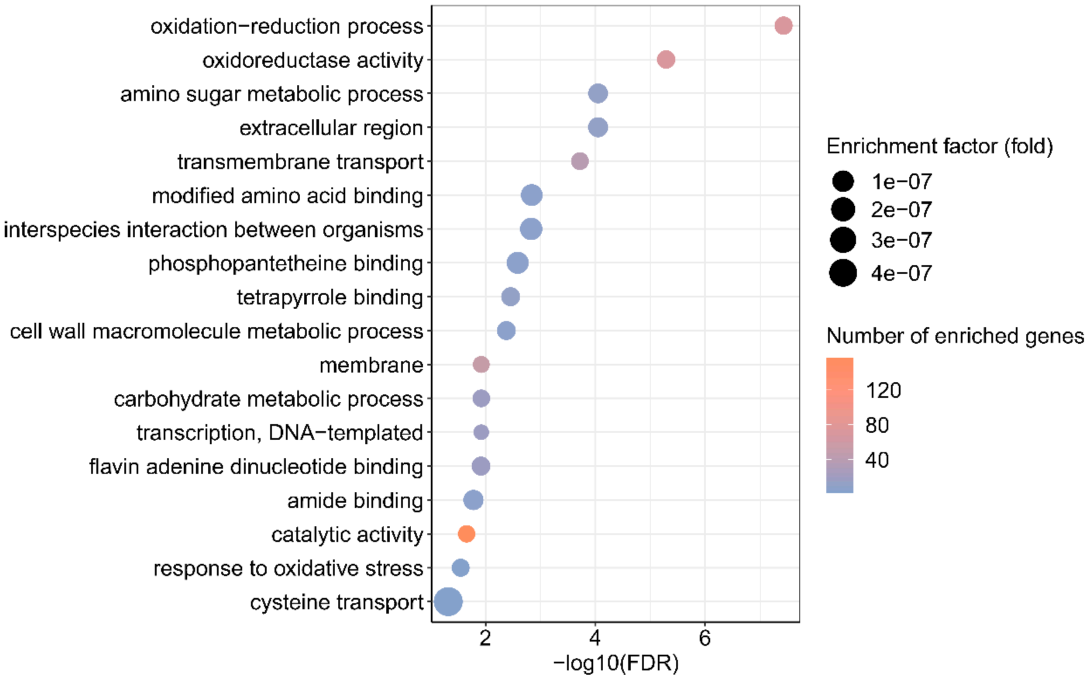

Figure S7. GO analysis of down-regulated genes in the 12-hours hyphae of the wild-type strain PH-1 and the *Fgsfl1* mutant.

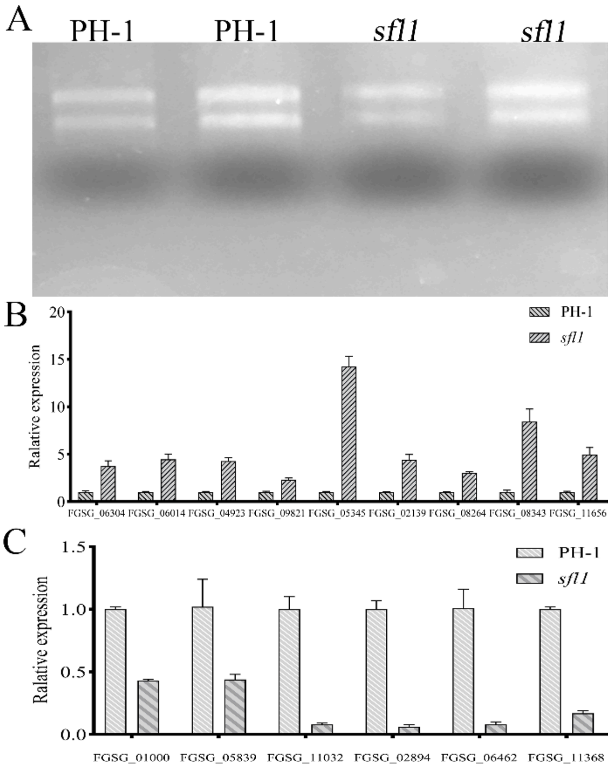

Figure S8. qRT-PCR analysis of the wild-type stain PH-1 and the *Fgsfl1* mutant. **A.** RNA gel detection of PH-1 and *sfl1* mutant. **B.** Up-regulation gene expression in the *sfl1* mutant in comparison with the wild type. **C.** Down-regulation gene expression in the *sfl1* mutant in comparison with the wild type.
