# Supplementary figures and images for: FgSfl1 and Its Conserved PKA Phosphorylation Sites Are Important for Conidiation, Sexual Reproduction, and Pathogenesis in Fusarium graminearum
Source: J Fungi (Basel). 2021 Sep 14;7(9):755. doi: 10.3390/jof7090755 (PMC8466192; doi:10.3390/jof7090755)

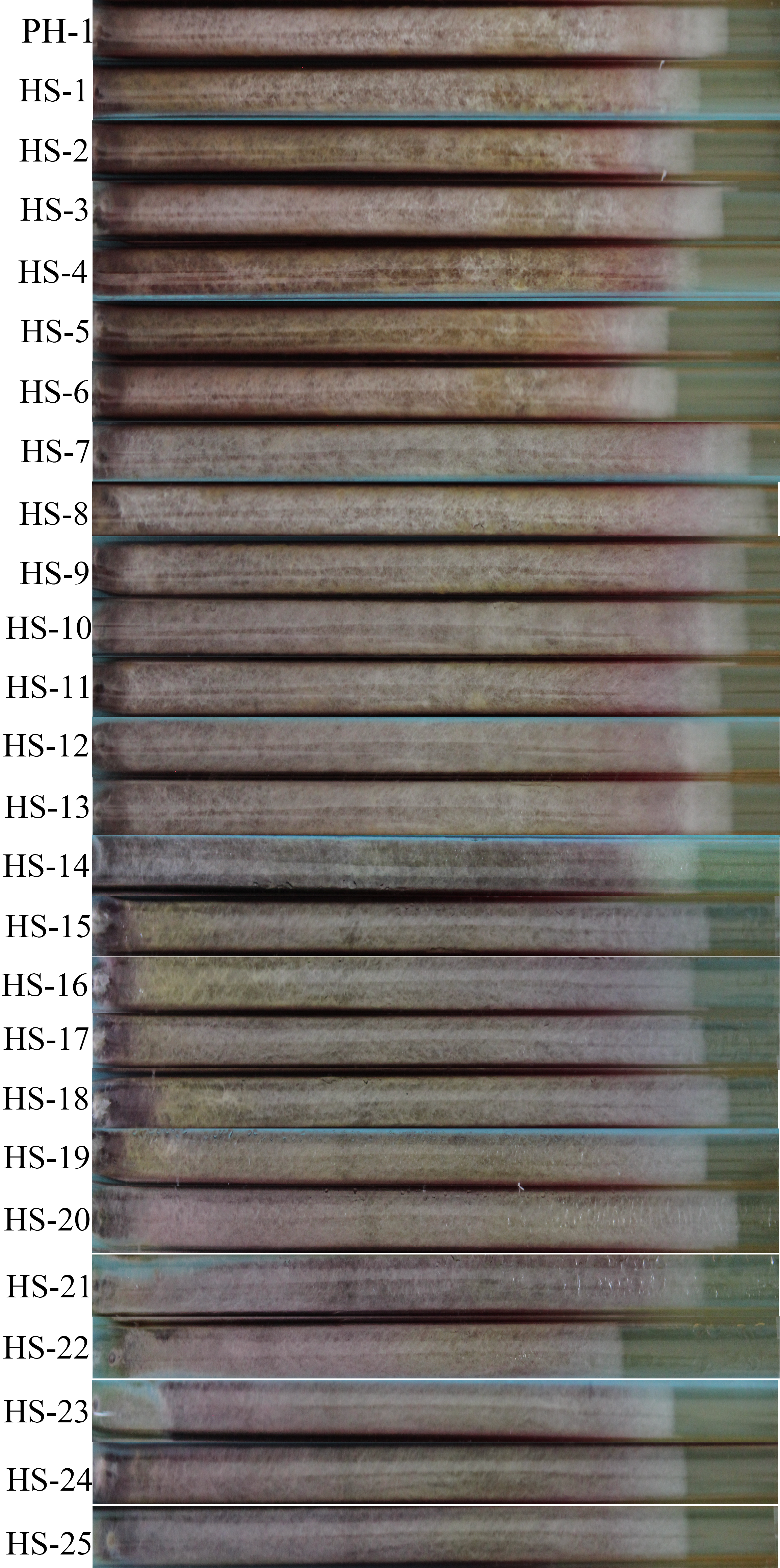

Supplement: Supplementary file 1 [file jof-07-00755-s001.zip › Fig.S1.tif]

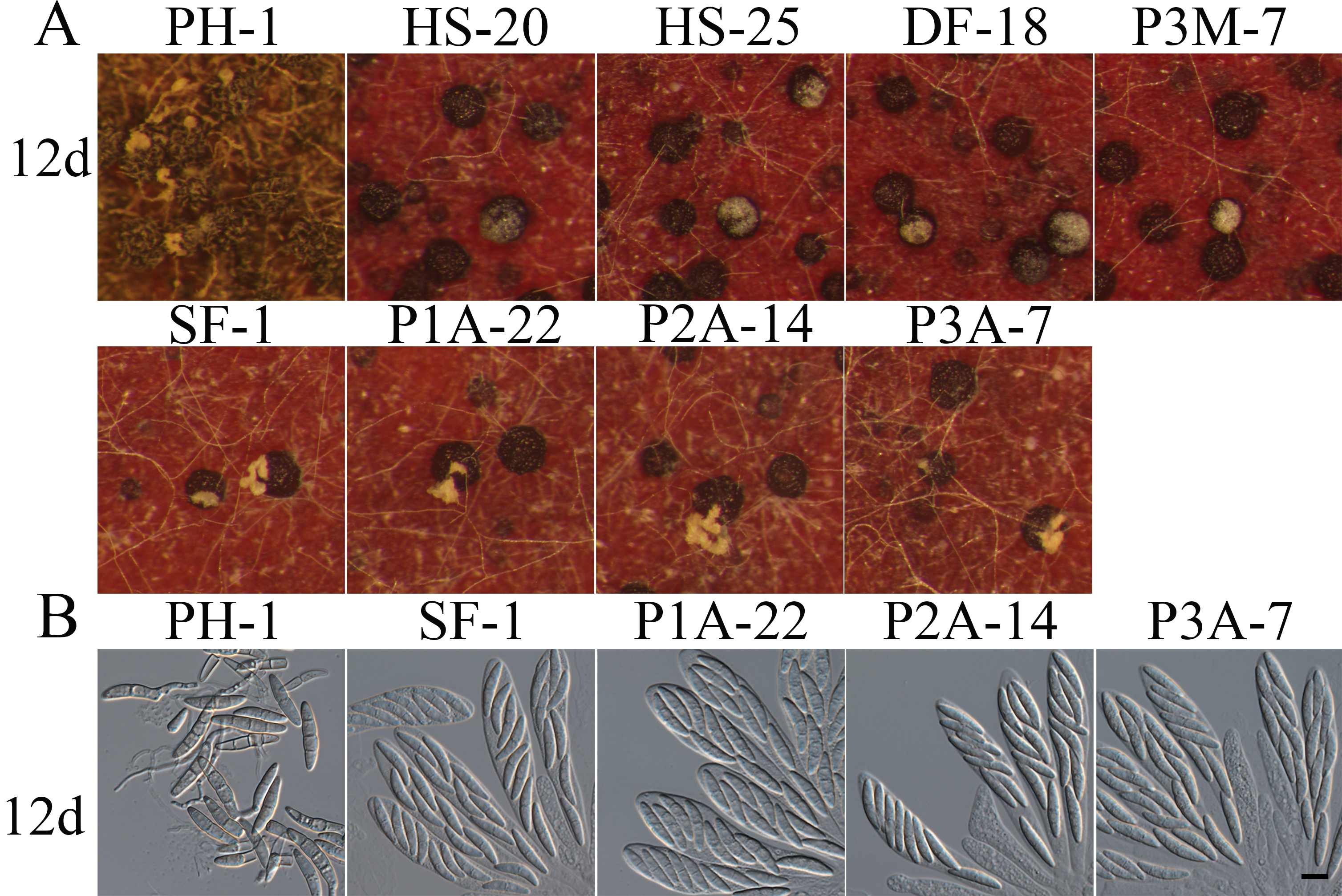

Supplement: Supplementary file 1 [file jof-07-00755-s001.zip › Fig.S2.tif]

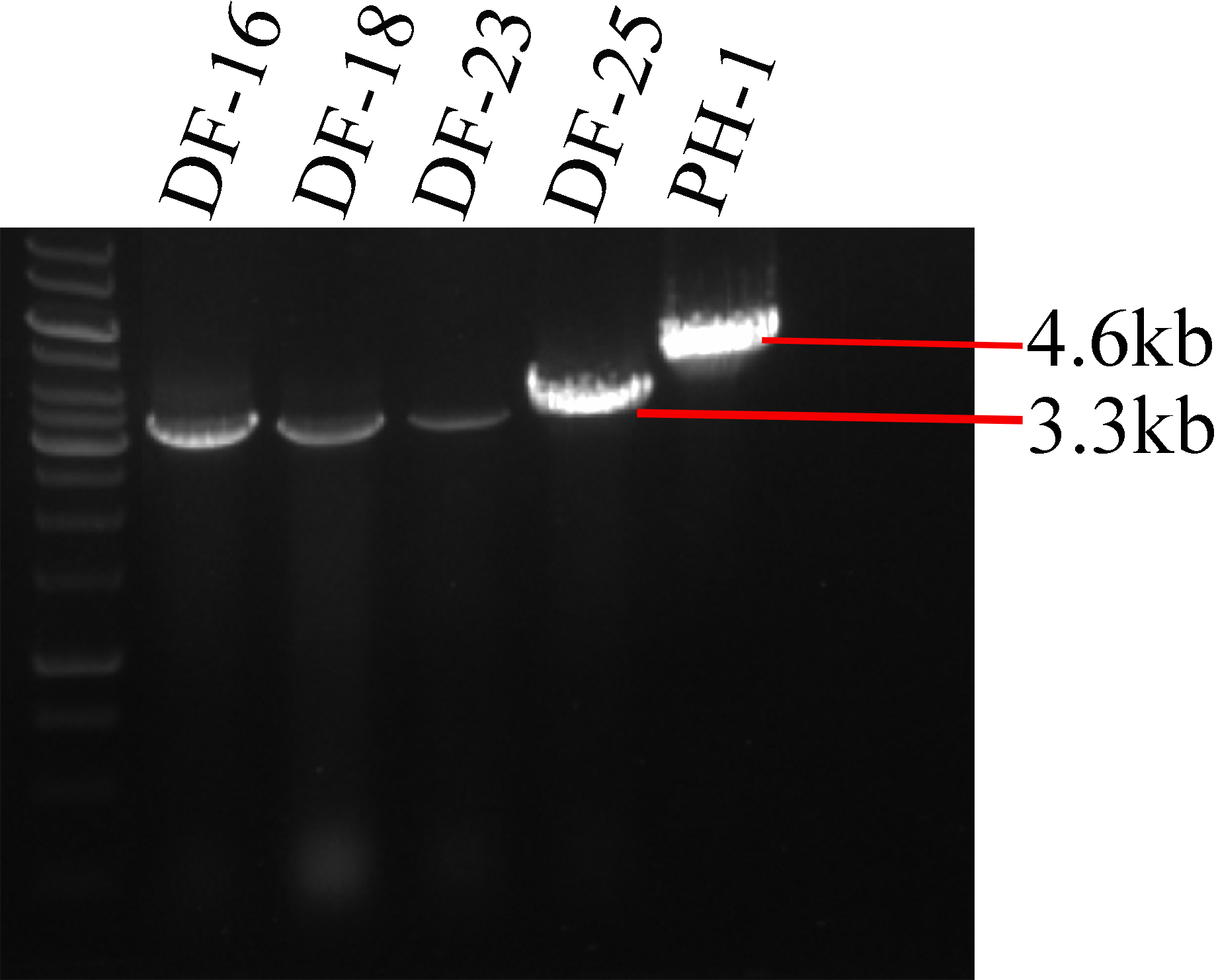

Supplement: Supplementary file 1 [file jof-07-00755-s001.zip › Fig.S3.tif]

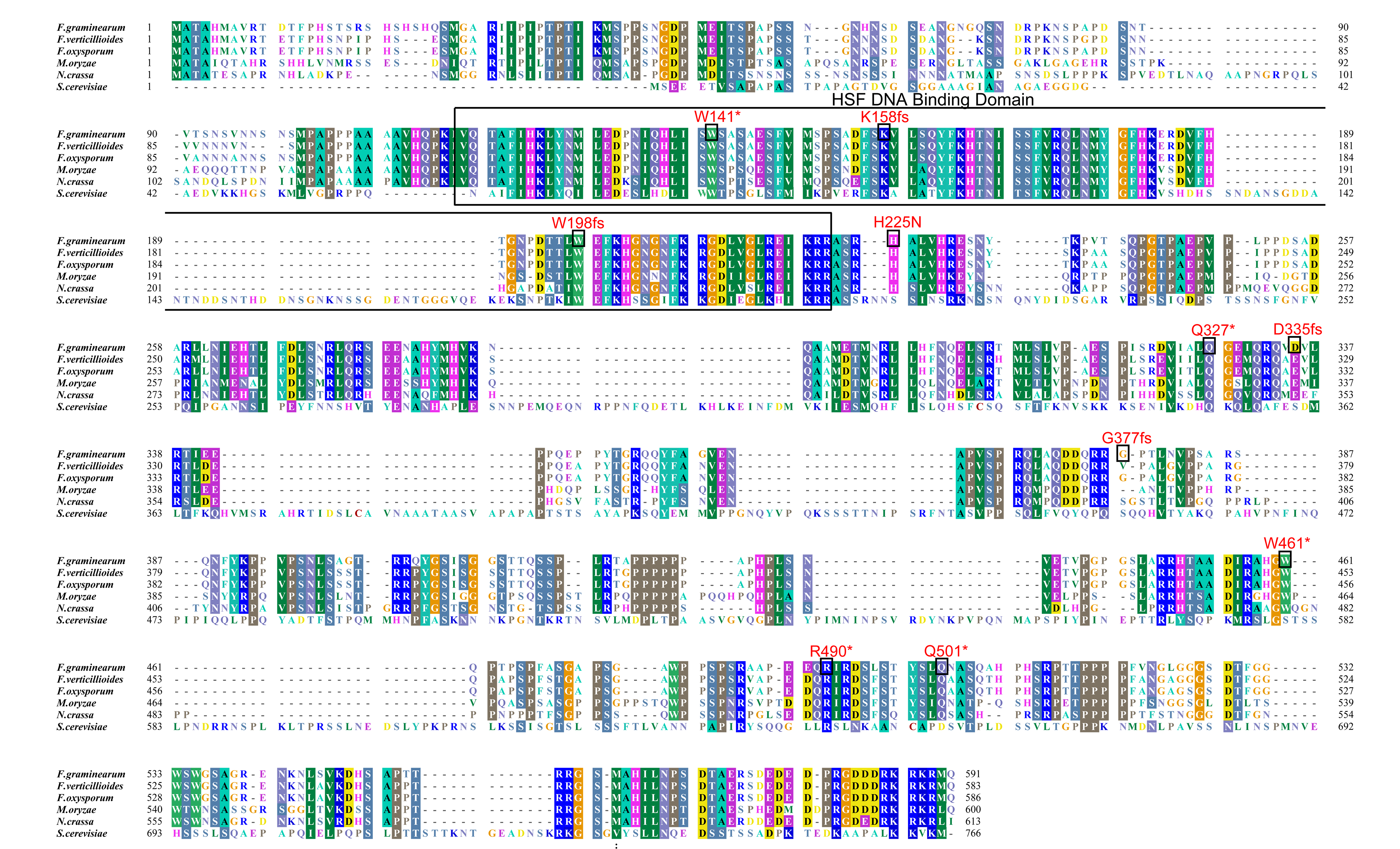

Supplement: Supplementary file 1 [file jof-07-00755-s001.zip › Fig.S4.tif]

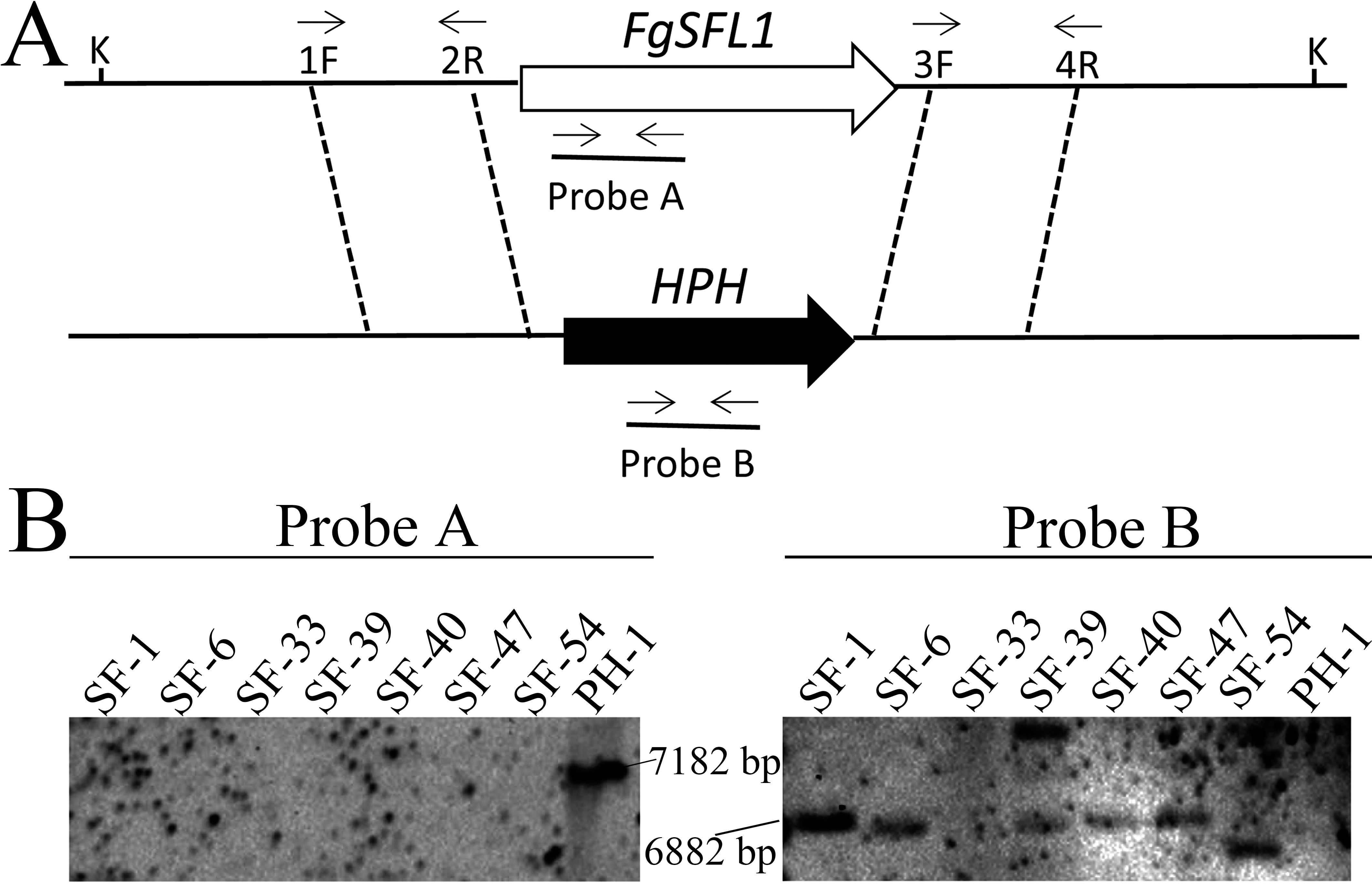

Supplement: Supplementary file 1 [file jof-07-00755-s001.zip › Fig.S5.tif]

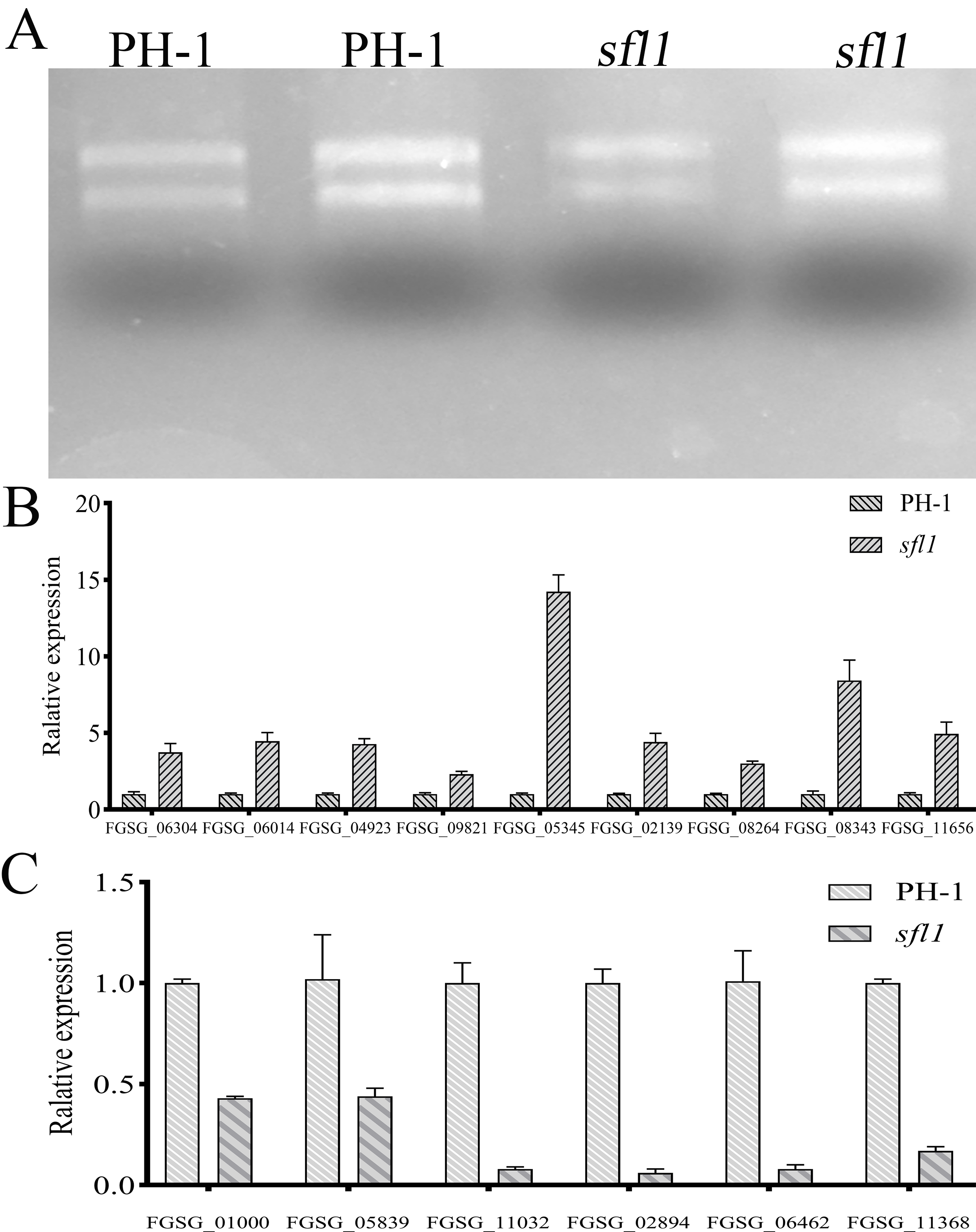

Supplement: Supplementary file 1 [file jof-07-00755-s001.zip › Fig.S8.tif]
